# Characterization of *O*-acetylation in sialoglycans by MALDI-MS using a combination of methylamidation and permethylation

**DOI:** 10.1038/srep46206

**Published:** 2017-04-07

**Authors:** Zhaoguan Wu, Henghui Li, Qiwei Zhang, Xin Liu, Qi Zheng, Jianjun Li

**Affiliations:** 1 Key Laboratory of Optoelectronic Chemical Materials and Devices of Ministry of Education, Institute for Interdisciplinary Research, School of Chemical and Environmental Engineering, Jianghan University, Wuhan, 430056 China; 2 College of Life Science and Technology, Huazhong University of Science and Technology, Wuhan, 430074 China; 3 Human Health Therapeutics, National Research Council Canada, 100 Sussex Drive, Ottawa, ON, K1A 0R6 Canada

## Abstract

*O*-Acetylation of sialic acid in protein N-glycans is an important modification and can occur at either 4-, 7-, 8- or 9-position in various combinations. This modification is usually labile under alkaline reaction conditions. Consequently, a permethylation-based analytical method, which has been widely used in glycomics studies, is not suitable for profiling *O*-acetylation of sialic acids due to the harsh reaction conditions. Alternatively, methylamidation can be used for N-glycan analysis without affecting the base-labile modification of sialic acid. In this report, we applied both permethylation and methylamidation approaches to the analysis of *O*-acetylation in sialic acids. It has been demonstrated that methylamidation not only stabilizes sialic acids during MALDI processing but also allow for characterization of their *O*-acetylation pattern. In addition, LC-MS/MS experiments were carried out to distinguish between the *O*-acetylated glycans with potential isomeric structures. The repeatability of methylamidation was examined to evaluate the applicability of the approach to profiling of *O*-acetylation in sialic acids. In conclusion, the combination of methylamidation and permethylation methodology is a powerful MALDI-TOF MS-based tool for profiling *O*-acetylation in sialic acids applicable to screening of N-glycans.

Glycosylation is a universal post-translational modification of proteins in eukaryotic species and plays key roles in protein folding, protein-protein interaction, cell-cell recognition, cancer metastasis, and the immune system^1^,^2^,^3^,^4^. In protein N-linked glycans, sialic acids, usually found as terminal monosaccharides, are the most important monosaccharides for human evolution^5^. Sialic acids are a family of 9-carbon carboxylated sugars, in which the most common one is *N*-acetyl-neuraminic acid (2-keto-5-acetamido-3,5-dideoxy-D-glycero-D-galactononulopyranos-1-onic acid) (Neu5NAc)^6^. Neu5NAc can be further modified, of which *O*-acetylation is one of the major modifications that significantly alters biological properties of the parent molecule. *O*-Acetylation can occur at either 4-, 7-, 8-, or 9-hydroxyl position^7^,^8^ and is regulated in a molecule specific, tissue-specific, and developmentally regulated fashion^9^,^10^,^11^,^12^,^13^,^14^,^15^. *O*-Acetyl groups may cause conformational change of glycoproteins and reduce the hydrophilic properties of sialic acids^16^. Sialic acid acetylesterase has a strong genetic link to susceptibility in relatively common human autoimmune disorders^17^. *N*-glycolylneuraminic acid (Neu5Gc) is a sialic acid molecule found in most non-human mammals and closely related to Neu5NAc. Neu5Gc is highly and selectively enriched in red meat, such as beef and pork. It can be metabolically incorporated into human tissues from dietary sources. Moreover, it was found that the interactions of Neu5Gc antigen with circulating anti-Neu5Gc antibodies in human body could potentially induce inflammation^18^.

Electrospray ionization (ESI) and matrix-assisted laser desorption/ionization (MALDI) mass spectrometry (MS) have been widely used in glycomics studies^19^,^20^,^21^,^22^,^23^,^24^,^25^. Using capillary electrophoresis-ESI-MS, we investigated the changes of *O*-acetylation pattern in sialic acids in N-glycans of salmon (*Salmo salar*) serum^26^,^27^. The analysis of native N-glycans revealed that the *O*-acetylation pattern was correlated with long-term handling stress, which was most likely to be a product of immune reaction. With the permethylation process, MALDI-MS is the desired technique for profiling glycans^28^,^29^,^30^,^31^. Methylation of all hydroxyl groups of glycans can significantly increase detection sensitivity because the derivatives are considerably more hydrophobic and stable than native glycans. In addition, permethylated glycans produce more information in tandem mass spectra that is very useful for linkage analysis. Unfortunately, permethylation process removes acetyl esters, which precludes its application to the characterization of *O*-acetylation of sialic acids. So far, detailed *O*-acetylation profiles and biological roles of these substitutions have been overlooked because of their lability to conventional purification and detection methods. An alternative strategy for stabilizing a sialic acid is the selective modification of its carboxyl group, including methylamidation^32^,^33^,^34^,^35^, dimethylamidation^19^, methyl esterification^36^ and ethyl esterification^37^,^38^.

This study aims at the development of MALDI-TOF MS for glycomics, especially the profiles of *O*-acetylation in sialic acids. To demonstrate the applicability of the proposed method, we analysed sera from seven different species of carp, including crucian carp, grass carp, silver carp, bighead carp, common carp, bream and black carp from wild fisheries^39^. The enzymatically released and purified fish serum N-glycans were subjected to methylamidation and permethylation derivatization and analysed using MALDI-TOF MS and LC-MS/MS. The tandem mass spectrometry experiments were performed to characterize glycan structures.

## Results and Discussion

### Crucian carp serum

**Figure 1:**
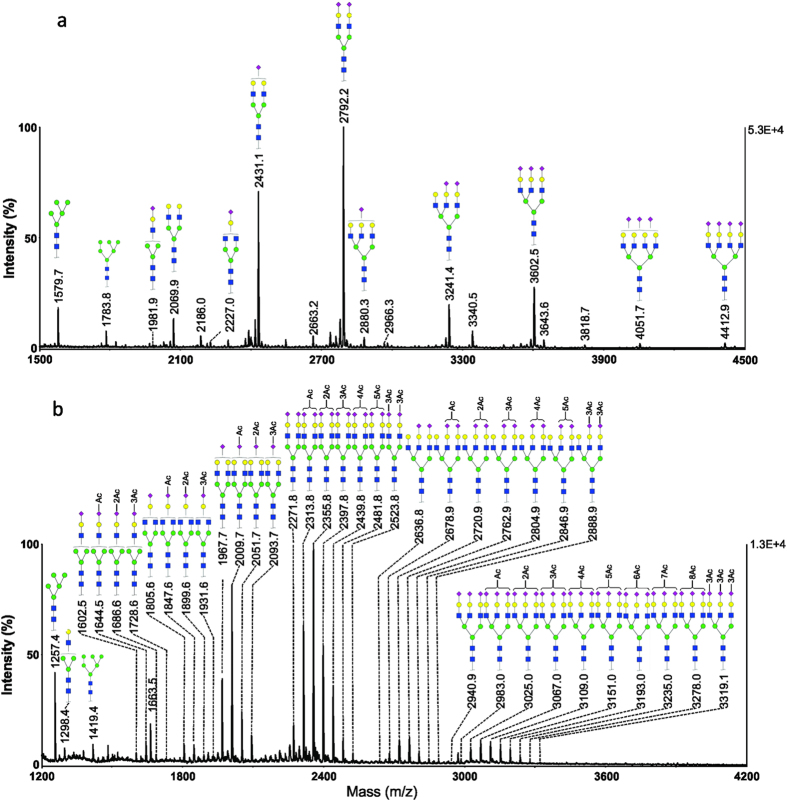
MALDI-MS analysis of N-glycans from a representative serum sample of crucian carp (Crucian-1). Monosaccharides represented as galactose (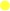), mannose (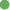), *N*-acetylglucosamine (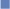), *N*-acetylneuraminic (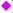) and fucose (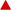). (**a**) MS spectrum of permethylated N-glycans; (**b**) MS spectrum of methylamidated N-glycans.

Permethylation reaction removes O-linked modification, thus the resultant MALDI MS spectrum can be significantly simplified and used to derive the composition of N-glycans. A representative MALDI-MS spectrum of permethylated N-glycans from crucian carp (*Carassius carassius*) serum sample is presented in Fig. 1a. For simplicity, this serum sample was labelled as Crucian-1. The primary structures of N-glycans isolated from crucian carp serum are similar to that of salmon serum^26^,^27^. Four major glycoforms, *m*/*z* 2431.1, 2792.2, 3241.4 and 3602.5, correspond to one, two and three sialic acid-containing oligosaccharides with chemical compositions of Neu5NAc_1_Hex_5_HexNAc_4_, Neu5NAc_2_Hex_5_HexNAc_4_, Neu5NAc_2_Hex_6_HexNAc_5_, and Neu5NAc_3_Hex_6_HexNAc_5_, respectively. Two high mannose structures were detected at *m/z* 1579.7 (Hex_5_HexNAc_2_) and *m/z* 1783.8 (Hex_6_HexNAc_2_). The non-sialylated biantennary structure was detected at *m/z* 2069.9 with a composition of Hex_5_HexNAc_4_. The detection of ions at *m/z* 4051.7 and 4412.9 suggests the presence of tetra-antennary structures with compositions of Neu5NAc_3_Hex_7_HexNAc_6_ and Neu5NAc_4_Hex_7_HexNAc_6_, respectively.

The MALDI-TOF MS spectrum of methylamidated N-glycans from crucian carp serum sample (Crucian-1) is shown in Fig. 1b. As expected, the methylamidation process completely derivatized all sialic acids and preserved the labile modifications, *i.e. O*-acetyl groups in sialic acids. The spectrum revealed several clusters of ions, with 42 Da intervals within each cluster. This observation suggests that one sialic acid can be modified with up to three *O*-acetyl groups. For example, the ions at *m/z* 1967.7, 2009.7, 2051.7 and 2093.7 may be assigned to monosialylated glycoforms with addition of zero, one, two and three *O*-acetyl groups, respectively. For the glycans containing two sialic acid residues, the ions at *m/z* 2271.8, 2313.8, 2355.8, 2397.8, 2439.8, 2481.8 and 2523.8 correspond to compositions of Neu5NAc_2_Hex_5_HexNAc_4_, OAc_1_Neu5NAc_2_Hex_5_HexNAc_4_, OAc_2_Neu5NAc_2_Hex_5_HexNAc_4_, OAc_3_Neu5NAc_2_Hex_5_HexNAc_4_, OAc_4_Neu5NAc_2_Hex_5_HexNAc_4_, OAc_5_Neu5NAc_2_Hex_5_HexNAc_4_, OAc_6_Neu5NAc_2_Hex_5_HexNAc_4_, respectively. Overall, up to six *O*-acetyl groups were detected for the N-glycans containing two sialic acid residues. This observation reveals the most extensively *O*-acetylated N-glycans in any fish species that have been reported so far^26^,^27^.

*O*-acetylation of sialic acid can occur at four positions, *i.e.* the 4-, 7-, 8-, or 9-hydroxyl position^7^,^8^. For any disialylated N-glycan containing six *O*-acetyl groups, two isomers may exist. Each of two sialic acids has either three *O*-acetyl groups (3 + 3) or one has four and the other one has two *O*-acetyl groups (4 + 2). Therefore, it is necessary to perform tandem mass spectrometry to characterize the distribution of *O*-acetyl groups. We performed LC-MS/MS experiments on the methylamidated sample and the representative MS/MS spectra are shown in Figure S1 in Supporting Information. For non *O*-acetylated glycans, the prominent fragment ions at *m/z* 305.1 and *m/z* 287.1 correspond to the methylamidated Neu5NAc (addition of 13 Da) and its anhydrous form (Figure S1a in Supporting Information). The non *O*-acetylated parent ions also afforded a Neu5NAc-containing fragment ion at *m/z* 670.1, corresponding to Neu5NAc_1_Hex_1_HexNAc_1_. When one of two sialic acids was *O*-acetylated, the tandem MS spectrum showed two fragment ions with a difference of 42 Da at *m/z* 670.3 and *m/z* 712.3 (Figure S1b in Supporting Information). The intensities of two fragment ions associated with trisaccharide residues, Neu5NAc_1_Hex_1_HexNAc_1_ and OAc_1_Neu5NAc_1_Hex_1_HexNAc_1_, were almost identical. However, the intensities of the fragment ions that corresponded to the monosaccharide residues, Neu5NAc and OAc_1_Neu5NAc, exhibited significant difference (*m/z* 305.1 and *m/z* 347.1 in Figure S1b in Supporting Information). For the glycans containing two *O*-acetyl groups, the predominant fragment ion at *m/z* 712.3 revealed that each sialic acid contained one *O*-acetyl group (1 + 1) rather than the combination of two *O*-acetyl groups and no *O*-acetyl modification (2 + 0). For the glycans containing three *O*-acetyl groups, the major species were 2 + 1 (*m/z* 712.3 and *m/z* 754.3) distribution, while the combination of 3 + 0 was only a minor species (*m/z* 796.3 and *m/z* 670.3) (Figure S1c in Supporting Information). For the glycans with six *O*-acetyl groups, the tandem MS spectrum demonstrated that each sialic acid was modified with three *O*-acetyl groups (Figure S1f in Supporting Information), which implied that the number of *O*-acetyl modification for a sialic acid was not higher than 3. We also noted that the LC-MS/MS experiments on the glycans containing multi sialic acids did not produce any fragments that could be correlated to the presence of disialic acid residues, *i.e.* Neu5NAc-Neu5NAc.

To study the biological variation, N-glycans from two additional individual fish serum samples, labelled as Crucian-2 and Crucian-3, were analysed and the obtained mass spectra of the replicate serum samples are presented in Figure S2 in Supporting Information. Although the relative abundance of *O*-acetylated species varied, the maximum numbers of *O*-acetyl groups for disialylated N-glycan remained unchanged among the three individual fish. The detected ions and the proposed compositions are summarized in Table S1 in Supporting Information.

### Repeatability of methylamidation

**Figure 2:**
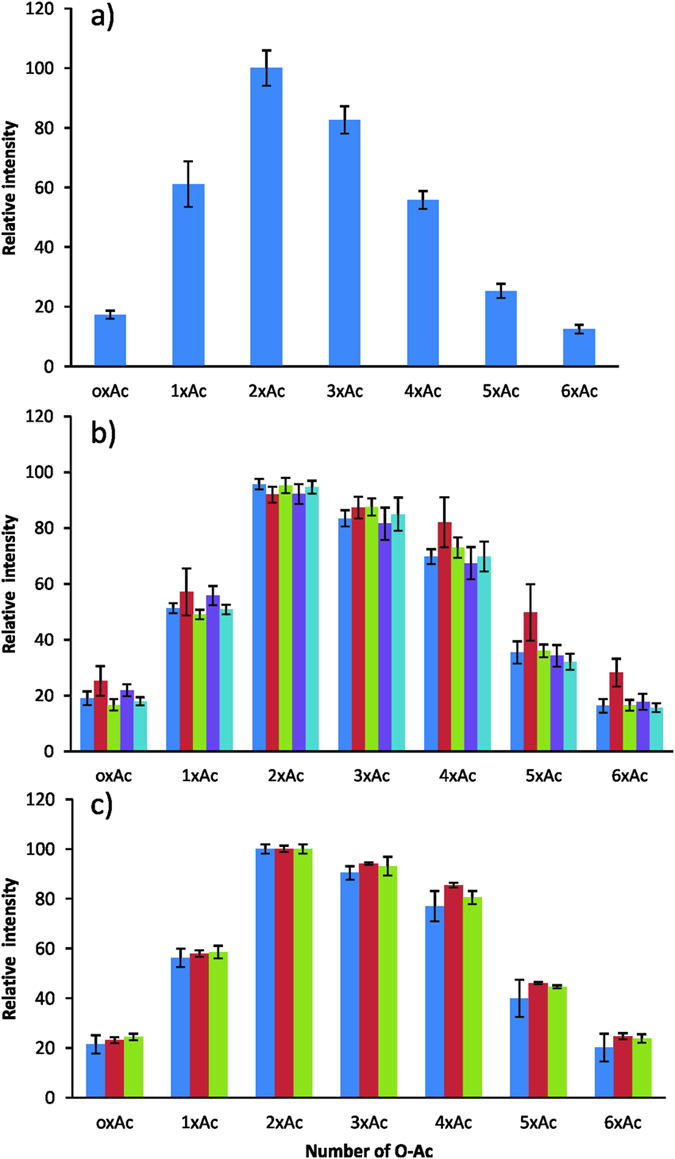
Repeatability of profiling *O*-acetylation in sialic acids by methylamidation for Neu5NAc_2_Hex_5_HexNAc_4_. The graph shows the average relative intensities of OAc_0-6_Hex_5_HexNAc_4_Neu5NAc_2_, with error bars for standard deviation. (**a**) Five methylamidated samples were prepared from the pooled serum in the same day; (**b**) The day-to-day repeatability analysis was performed for three different days; (**c**) Native glycans were measured by ESI-MS.

Methylamidation has been previously proven to be an effective derivatization strategy for sialic acid-containing N-glycans^32^,^33^,^34^,^35^. Complete methylamidation of 2,6-sialyllactose could be achieved within 10 min, whereas the complete reaction for 2,3-sialyllactose took about 30 min^32^. So far, the applicability of methylamidation to profiling *O*-acetylation patterns of sialic acids has not been investigated. Because no *O*-acetylated N-glycan standards were available, we used the N-glycans from pooled serum samples of multiple crucian carp for repeatability testing. The glycans Neu5NAc_2_Hex_5_HexNAc_4_ with different *O*-acetyl groups were selected to investigate the stability of *O*-acetyl groups, by comparing ESI-MS analysis of native glycans and MALDI-MS analysis of methylamidated glycans. The intensities of native glycans from 5 technical replicates in ESI-MS experiments were summed and normalized to the sum of intensities of the highest peak (Fig. 2a). To investigate the stability of *O*-acetyl groups under methylamidation conditions, 5 samples from serum pool were individually PNGase-F digested and derivatized, followed by MALDI-MS analysis. The intensities of each glycan from 3 spots in MALDI target were summed and normalized to the sum of intensities of the highest peak (*i.e.* the glycan containing three *O*-acetyl groups). The average relative intensity values for six glycoforms are presented in Fig. 2b, with the CVs ranging from 1.9% to 25.4%. We then derivatized 5 N-glycan samples from the same serum pool collected on each day for two additional days to evaluate the day-to-day repeatability^38^. The relative intensity values of six glycoforms for three different days are illustrated in Fig. 2c, with the CVs ranging from 0.8 to 27.8%. While the profiles between native glycans (ESI-MS) and their methylamine derivatives (MALDI-MS) were similar, relatively lower intensities in ESI-MS spectra were observed for the glycans containing four, five and six *O*-acetyl groups, respectively. To further demonstrate the applicability of the approach, we analysed N-glycans from serum samples of additional six fish species.

### Common carp serum

**Figure 3:**
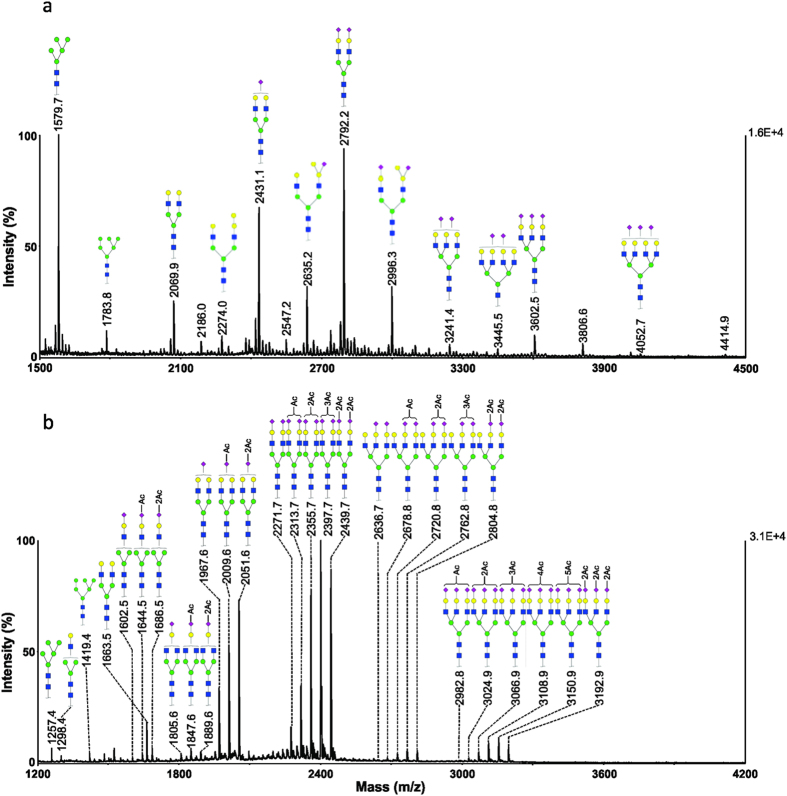
MALDI-MS analysis of N-glycans from a representative serum sample of common carp (Common-1). (**a**) MS spectrum of permethylated N-glycans; (**b**) MS spectrum of methylamidated N-glycans.

The MALDI-TOF MS spectra of permethylated and methylamidated N-glycans from a representative serum sample of common carp (*Cyprinus carpiois*), labelled as Common-1, are shown in Fig. 3. Two major types of glycans were detected at *m*/*z* 2431.1 and 2792.2, corresponding to mono- and disialylated N-glycans with chemical compositions of Neu5NAc_1_Hex_5_HexNAc_4_ and Neu5NAc_2_Hex_5_HexNAc_4_, respectively. Similarly to sera from crucian carp, common carp serum also expressed two high mannose structures, *i.e.* Hex_5_HexNAc_2_ (*m/z* 1579.7) and Hex_6_HexNAc_2_ (*m/z* 1783.8).

The methylamidation analysis indicated that up to two *O*-acetyl groups were attached to one sialic acid. MS/MS experiments were also carried out and the representative MS/MS spectra are presented in Figure S3 in Supporting Information. The monosialylated glycan at *m/z* 1967.6 corresponds to a non *O*-acetylated species; whereas ions at *m/z* 2009.6 and 2051.6 correspond to the addition of one and two acetyl groups, respectively. The glycan containing two sialic acids could be modified with up to four *O*-acetyl groups, *i.e. m/z* 2313.7, 2355.7, 2397.7, and 2439.7, corresponding to compositions OAc_1_Neu5NAc_2_Hex_5_HexNAc_4_, OAc_2_Neu5NAc_2_Hex_5_HexNAc_4_, OAc_3_Neu5NAc_2_Hex_5_HexNAc_4_ and OAc_4_Neu5NAc_2_Hex_5_HexNAc_4_, respectively. However, no glycans containing tri-*O*-acetyl groups were found in common carp serum samples. Larger glycans with triantennary structures were also detected, containing two or three sialic acids. For disialylated glycans, the ions detected at *m/z* 2678.8, 2720.8, 2762.8 and 2804.8 corresponded to the compositions of OAc_1_Neu5NAc_2_Hex_6_HexNAc_5_, OAc_2_Neu5NAc_2_Hex_6_HexNAc_5_, OAc_3_Neu5NAc_2_Hex_6_HexNAc_5_ and OAc_4_Neu5NAc_2_Hex_6_HexNAc_5_, respectively. Trisialylated glycans were found to be modified with up to six *O*-acetyl groups, i.e. *m/z* 3024.9 (2xOAc), 3066.9 (3xOAc), 3108.9 (4xOAc), 3150.9 (5xOAc) and 3192.9 (6xOAc).

The MALDI-MS spectra for the N-glycans from serum samples of two additional individual common carp, labelled as Common-2 and Common-3, are presented in Figure S4 in Supporting Information. The *O*-acetylation pattern in N-glycans from Common-2 (Figure S4b and Figure S4e) was similar to that shown in Common-1 (Figure S4a and Figure S4d). However, *O*-acetylation pattern in N-glycans from Common-3 showed much lower degree of *O*-acetylation (Figure S4c and Figure S4f), in which only four and six *O*-acetyl groups were detected in disialylated glycans and trisialylated glycans, respectively. The detected ions and their corresponding compositions are summarized in Table S2 in Supporting Information.

### Grass carp serum

**Figure 4:**
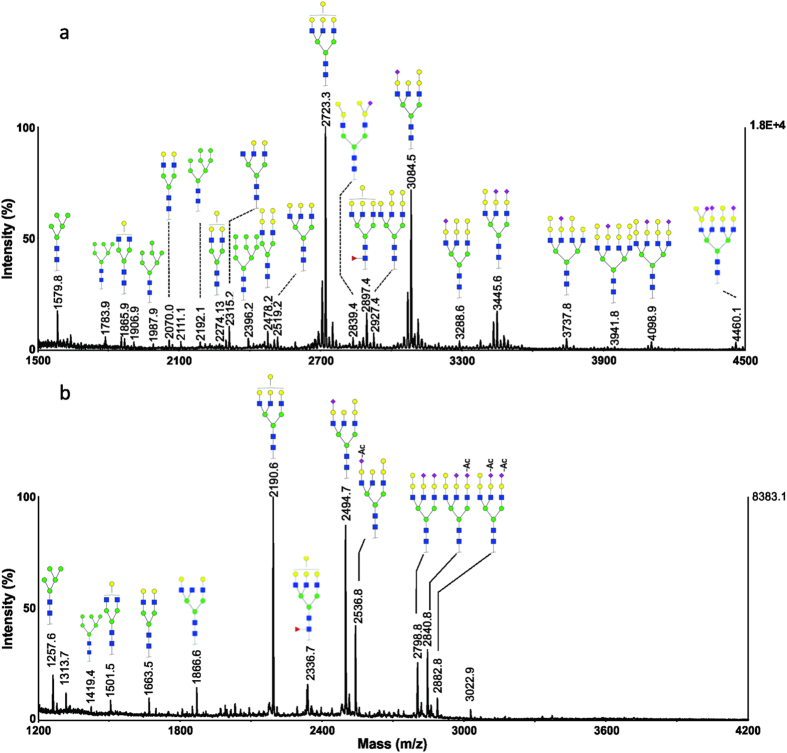
MALDI-MS analysis of N-glycans from a representative serum sample of grass carp (Grass-1). (**a**) MS spectrum of permethylated N-glycans; (**b**) MS spectrum of methylamidated N-glycans.

Three biological replicates of grass carp (*Ctenopharyngodon idella)* serum samples were analysed. The permethylated and methylamidated N-glycans profiles from a representative serum sample, labelled as Grass-1, are shown in Fig. 4, in which two major ions at *m/z* 2723.3 and 3084.5 correspond to triantennary oligosaccharides with chemical compositions of Hex_7_HexNAc_5_ and Neu5NAc_1_Hex_7_HexNAc_5_ (Fig. 4a). In the high mass region, the ions at *m/z* 3737.8, 3941.8, 4098.9 and 4460.1 correspond to tetraantennary oligosaccharides with different numbers of terminal Hex residues and sialic acid residues.

Methylamidation analysis was also carried out to explore the *O*-acetylation of N-glycans from the same grass carp serum sample. The resultant MALDI-MS spectrum is presented in Fig. 4b. The monosialylated glycan at *m/z* 2494.7 is a non *O*-acetylated glycan; whereas the ions at *m/z* 2536.8 correspond to the addition of one acetyl group with a chemical composition of OAc_1_Neu5NAc_1_Hex_7_HexNAc_5_. The disialylated structures at *m/z* 2798.8, 2840.8 and 2882.8 correspond to the glycans with addition of zero, one and two acetyl groups, respectively. The detected ions and their corresponding compositions for grass carp serum N-glycans are summarized in Table S3 and the representative MS/MS spectra are shown in Figure S5 in Supporting Information. These results indicate that the sialic acids in grass carp serum glycoproteins can only be modified with one *O*-acetyl group. The MALDI-MS spectra of serum N-glycans for three individual grass carp (*i.e.* Grass-1, Grass-2 and Grass-3) are presented in Figure S6 in Supporting Information. Similar *O*-acetylation patterns were observed in N-glycans from three biological replicates.

### Silver carp serum

**Figure 5:**
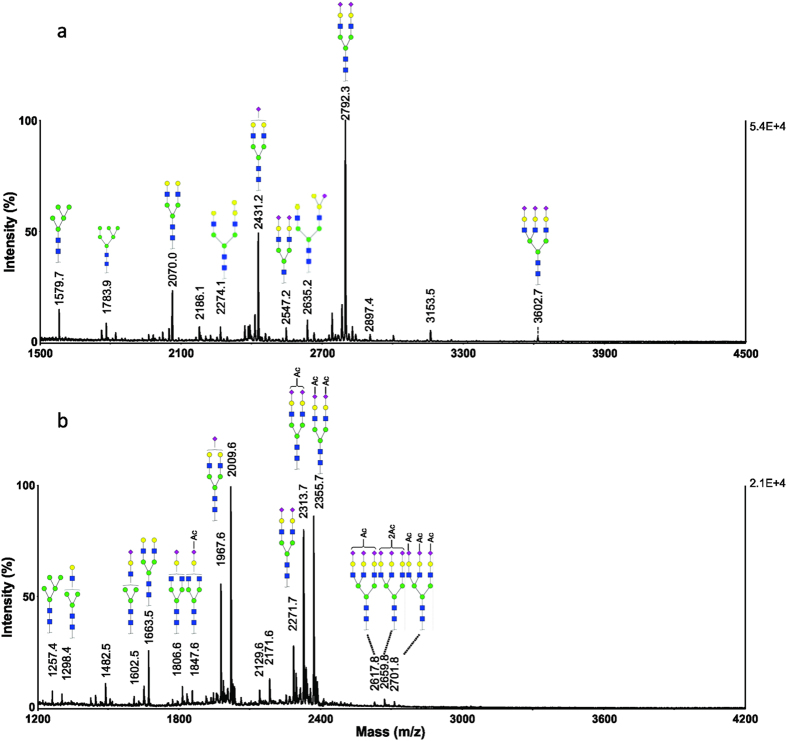
MALDI-MS analysis of N-glycans from a representative serum sample of silver carp (Silver-1). (**a**) MS spectrum of permethylated N-glycans; (**b**) MS spectrum of methylamidated N-glycans.

The permethylation and methylamidation analyses of N-glycans from a representative serum sample of silver carp (*Hypophthalmichthys molitrix*), labelled as Silver-1, are shown in Fig. 5. The most abundant glycans in permethylated spectrum are the biantennary structures at *m/z* 2070.0, 2431.2 and 2792.3 with addition of zero, one and two sialic acids, respectively. In the high mass region, the triantennary structures were also detected, *e.g. m/z* 3241.5 and 3602.7, with the chemical compositions of Neu5NAc_2_Hex_6_HexNAc_5_ and Neu5NAc_3_Hex_6_HexNAc_5_, respectively. The MALDI MS spectrum of methylamidated glycans is shown in Fig. 5b. The spectrum reveals several clusters of ions with 42 Da intervals within each cluster. For example, the ions detected at *m/z* 2271.7, 2313.7 and 2355.7 correspond to the biantennary glycoforms with zero, one and two *O*-acetyl groups, respectively. The triantennary trisialylated structures are almost exclusively modified with three *O*-acetyl groups. The representative MS/MS spectra are shown in Figure S7 in Supporting Information. Two additional biological replicates of silver carp serum samples were analysed and significant variations were observed between the three replicates. For example, the glycan with the composition of Neu5NAc_1_Hex_6_HexNAc_5_ (*m/z* 2880.3) was only detected in the MALDI MS spectra of permethylated glycans from Silver-2 (Figure S8b and Figure S8e in Supporting Information) or Silver-3 (Figure S8c and Figure S8f in Supporting Information), and the corresponding methylamidated glycans were detected at *m/z* 2332.9 (Neu5NAc_1_Hex_6_HexNAc_5_) and *m/z* 2374.9 (OAc_1_Neu5NAc_1_Hex_6_HexNAc_5_). Table S4 in Supporting Information summarizes the detected ions and their corresponding compositions.

### Bream carp serum

**Figure 6:**
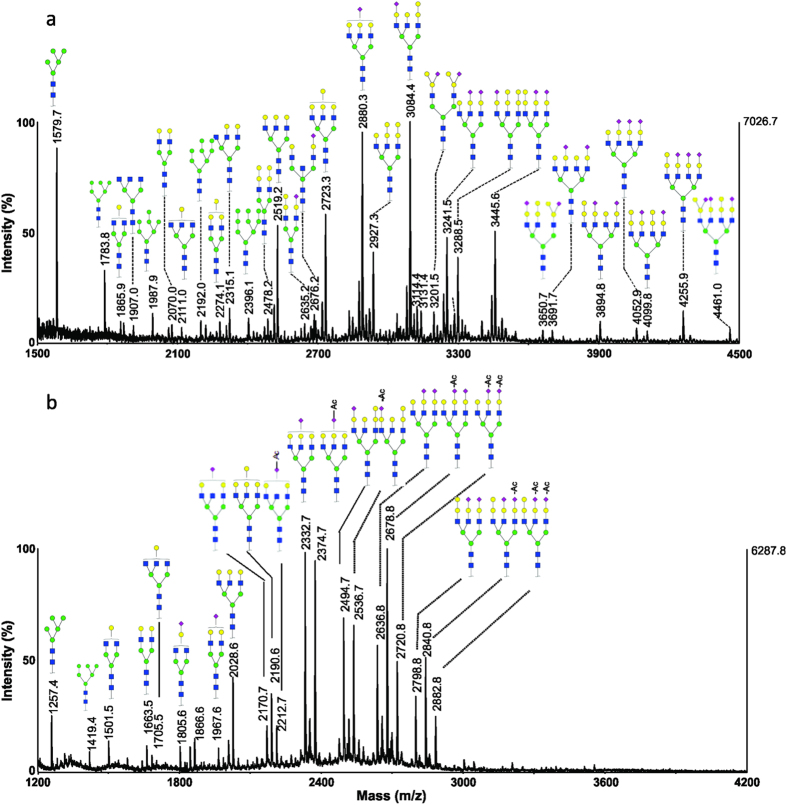
MALDI-MS analysis of N-glycans from a representative serum sample of bream carp (Bream-1). (**a**) MS spectrum of permethylated N-glycans; (**b**) MS spectrum of methylamidated N-glycans.

The N-glycans from a representative serum sample of bream carp, labelled as Bream-1, were initially analysed using permethylation method (Fig. 6a). The most abundant structures are the triantennary glycoforms with different numbers of sialic acids and Hex terminal motifs. For example, the ion detected at *m/z* 2723.3 is a triantennary structure with the chemical composition of Hex_7_HexNAc_5_. The ions at *m/z* 3084.4 correspond to the composition of Neu5NAc_1_Hex_7_HexNAc_5_, with the addition of a sialic acid group. More complex structures were observed as the tetraantennary glycans with ions at *m/z* 3894.8 and 4255.9, respectively. Methylamidation was used to investigate the *O*-acetyl modification in bream carp serum N-glycans as well (Fig. 6b). The monosialylated glycans at *m/z* 2212.7, 2374.7 and 2536.7 can be assigned to the addition of an *O*-acetyl group to the oligosaccharides with *m/z* 2170.8, 2332.7 and 2494.7, respectively. The disialylated glycans, with the addition of two *O*-acetyl groups, were detected at *m/z* 2720.8 (OAc_2_Neu5NAc_2_Hex_6_HexNAc_5_) and 2882.8 (OAc_2_Neu5NAc_2_Hex_7_HexNAc_5_). This observation suggests that the *O*-acetylation patterns of serum N-glycans from bream carp are similar for silver carp and grass carp. The representative MS/MS spectra are presented in Figure S9 in Supporting Information. Similar glycoforms were detected in two additional biological replicates (Bream-2 and Bream-3) as shown in Figure S10b, Figure S10c, Figure S10e and Figure S10f in Supporting Information, although relative intensities varied among the three replicates. The proposed compositions for most detected ions are presented in Table S5 in Supporting Information.

### Bighead carp serum

**Figure 7:**
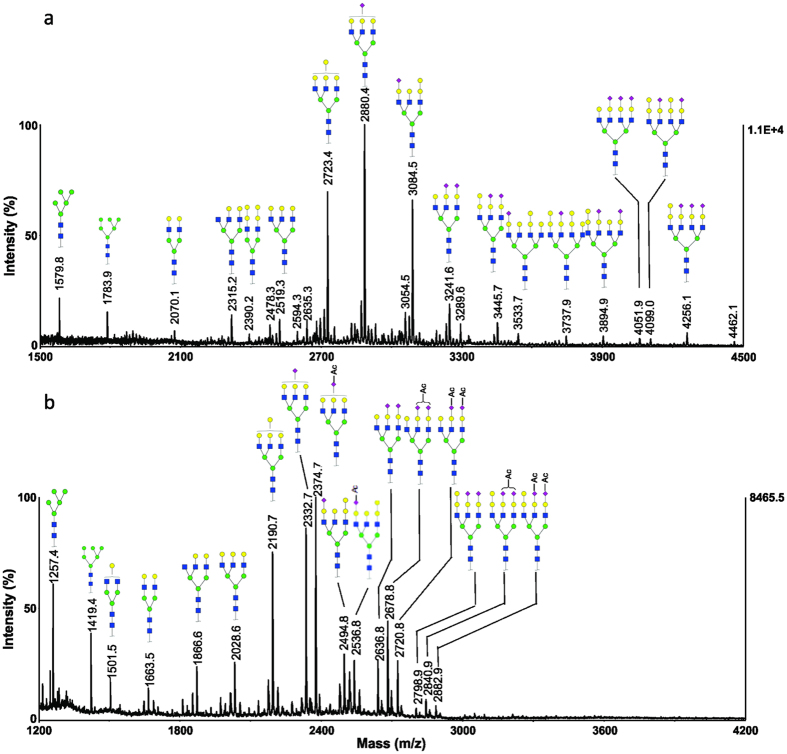
MALDI-MS analysis of N-glycans from a representative serum sample of bighead carp (Bighead-1). (**a**) MS spectrum of permethylated N-glycans; (**b**) MS spectrum of methylamidated N-glycans.

The N-glycan profiles of a representative serum sample from bighead carp (*Hypophthalmichthys nobilis*) were also investigated using both permethylation and methylamidation methods (Fig. 7). For permethylated glycans (Fig. 7a), the most abundant ions corresponded to triantennary glycoforms at *m/z* 2723.4, 2880.4 and 3084.4, which have the chemical compositions of Hex_7_HexNAc_5_, Neu5NAc_1_Hex_6_HexNAc_5_ and Neu5NAc_1_Hex_7_HexNAc_5_, respectively. High-mannose structures, *m/z* 1579.8 and 1783.9, were also detected. In the high mass region, several tetraantennary structures were observed, *e.g. m/z* 3533.7, 3894.9, 4051.9 and 4256.1, corresponding to different number of sialic acids and terminal Hex motifs.

The MS spectrum of methylamidated N-glycans illustrates the presence of *O*-acetyl modification in sialic acids (Fig. 7b). For example, the ion at *m/z* 2374.7 is the triantennary structure with one *O*-acetyl group (OAc_1_Neu5NAc_1_Hex_6_HexNAc_5_). The disialylated glycans at *m/z* 2636.8, 2678.8 and 2720.8 are with zero, one and two *O*-acetyl groups, *e.g.* Neu5NAc_2_Hex_6_HexNAc_5_, OAc_1_Neu5NAc_2_Hex_6_HexNAc_5_ and OAc_2_Neu5NAc_2_Hex_6_HexNAc_5_, respectively. It is worth noting that sialic acids in bighead carp serum sample have only one *O*-acetyl group, similarly to the *O*-acetylation found in grass carp, silver carp and bream carp serum glycans. Tandem mass spectrometry experiment confirmed the *O*-acetylation of sialic acids (Figure S11 in Supporting Information). The permethylated and methylamidated N-glycans of two additional replicate serum samples (*i.e.* Bighead-2 and Bighead-3) are shown in Figure S12b, Figure S12c, Figure S12e and Figure S12f in Supporting Information, respectively. The ion at *m/z* 3037.8 in the spectrum of permethylated glycans corresponded to a composition of Neu5NAc_2_Hex_5_HexNAc_5_. The methylamidated glycans with zero, one or two *O*-acetyl groups were detected at *m/z* 2474.9, 2516.9, and 2558.9, respectively. The detected ions and their compositions are summarized in Table S6 in Supporting Information.

### Black carp serum

**Figure 8:**
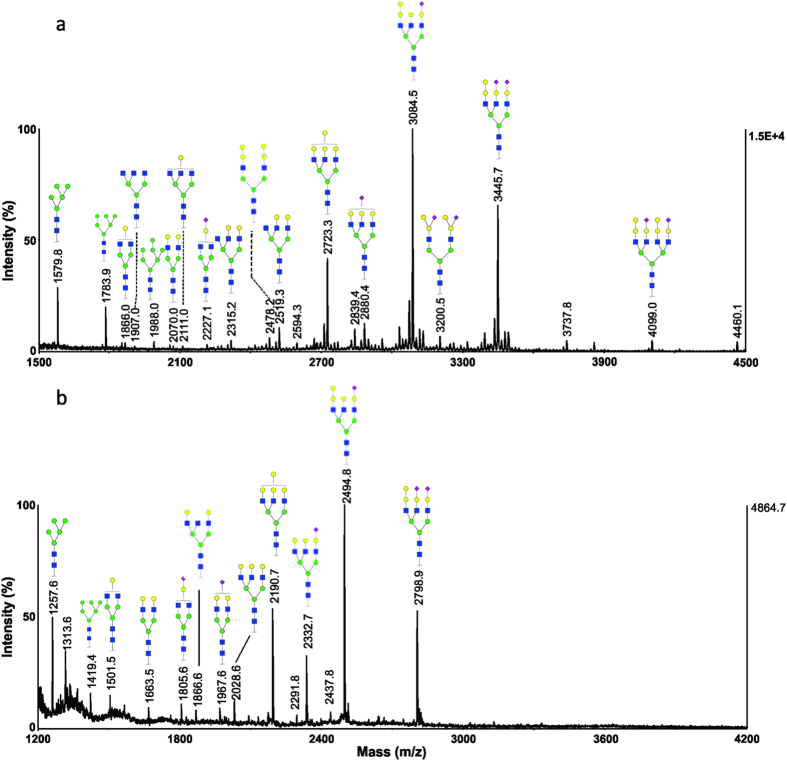
MALDI-MS analysis of N-glycans from a representative serum sample of black carp (Black-1). (**a**) MS spectrum of permethylated N-glycans; (**b**) MS spectrum of methylamidated N-glycans.

MALDI-MS profiling of the permethylated glycans released from a representative serum sample of black carp (*Mylopharyngodon piceus*), labelled as Black-1, indicated the presence of multi-antennary structures (Fig. 8a), which have been previously found in several different species of fresh-water fish and both unfertilized^40^,^41^ and fertilized fish eggs^42^,^43^,^44^. The most abundant glycan structures at *m*/*z* 2723.3, 3084.5 and 3445.7, correspond to the oligosaccharides with chemical compositions of Hex_7_HexNAc_5_, Neu5NAc_1_Hex_7_HexNAc_5_ and Neu5NAc_2_Hex_7_HexNAc_5_, respectively. The glycans from black carp serum sample were mainly found to exhibit complex tri- and tetra-antennary structures. Comparatively, the glycan compositions in crucian carp and common carp sera mainly exhibit biantennary structures and extensive *O*-acetyl modification. The N-glycans of black carp serum samples were also investigated using the methylamidation method (Fig. 8b). Surprisingly, no *O*-acetyl modification was detected for this species. The representative MS/MS spectra are shown in Figure S13 in Supporting Information. Two additional biological replicates (*i.e.* Black-2 and Black-3) confirmed the absence of *O*-acetylation in sialic acids of N-glycans from black carp serum samples (Figure S14 in Supporting Information). The relative abundance and type of each glycan in black carp serum samples are consistent between the permethylation and methylamidation spectra. The detected ions and their corresponding compositions are summarized in Table S7 in Supporting Information.

## Conclusion

In this study, we applied a new derivatization strategy, the combination of permethylation and methylamidation, for the characterization of *O*-acetylation of sialic acid in N-glycans. Methylamidation allows the stabilization of *O*-acetylated sialic acid residues; whereas permethylation provides a sensitive technique with simplified MALDI MS spectra that contain unambiguous information on glycan compositions. The serum samples from seven freshwater carp species were analysed, including crucian carp, grass carp, silver carp, common carp, bream and black carp. The results revealed that the N-glycans from sera of different fish species presented significant difference in composition, sialylation pattern and degree of *O*-acetylation. For example, the crucian carp serum exhibited up to three *O*-acetyl groups on a single sialic acid residue. The results also suggested that no more than three *O*-acetyl groups were attached to one sialic acid residue in all glycans studied, even in the ones with the most extensive *O*-acetyl substitution. While the method was developed for application in aquaculture, it is applicable for the analysis of sialoglycans derived from sera of any other animal species.

## Materials and Methods

### Chemicals and Materials

2,5-Dihydroxybenzoic acid (DHB), dimethyl sulfoxide (DMSO), sodium hydroxide, N-methylmorpholine, acetonitrile (ACN), methylamine hydrochloride, (7-azabenzotriazol-1-yloxy) trispyrrolidinophosphonium hexafluorophosphate (PyAOP), trifluoroacetic acid (TFA), 1-butanol, ethanol, porous graphitic carbon (PGC), microcrystalline cellulose (MCC) were obtained from Sigma-Aldrich (St. Louis, MO). Blood was collected from a local fish farm and the serum was separated by centrifugation and stored at −20 °C until used. *N*-Glycosidase F (PNGase F) and endoglycosidase buffer pack were purchased from New England Biolabs (Ipswich, MA, USA). Formic acid (FA), methyl iodide, chloroform, empty cartridges and frits were purchased from Aladdin (Shanghai, China). All solutions were prepared using deionized water purified by a Milli-Q purification system (Millipore, MA, USA).

### Preparation of N-glycans

Fish serum (10 μL) was dissolved in 50 μL of sodium phosphate (20 mM, pH 7.5) containing 0.2% SDS and 0.1 M DTT and denatured at 100 °C for 10 min. After cooling, 12 μL of 10% NP-40 and 38 μL of water were added. The reaction mixture was incubated with PNGase F (10 units) for 24 h at 37 °C. The sample was then boiled for 5 min to stop the reaction and the released glycans were purified using PGC cartridges. The first serum sample from crucian carp was labelled as Crucian-1 and similar definition was used for the first serum sample of other fish species, *i.e.* Common-1, Grass-1, Silver-1, Bream-1, Bighead-1 and Black-1.

Biological variation was established by analysing two additional replicates of serum samples for each fish species. Accordingly, the additional serum replicates for all species examined were abbreviated as Crucian-2 and Crucian-3, Common-2 and Common-3, Grass-2 and Grass-3, Silver-2 and Silver-3, Bream-2 and Bream-3, Bighead-2 and Bighead-3, Black-2 and Black-3. For comparison, the MALDI-TOF MS results for all three replicates of each species were included in Supporting Information, *i.e.* Figure S2, Figure S4, Figure S6, Figure S8, Figure S10, Figure S12 and Figure S14.

All experiments were performed in accordance with the protocols approved by Ethical Committee of Jianghan University.

### Purification of glycans

PGC cartridges were washed with 3.0 mL of 80% (v/v) ACN containing 0.1% TFA followed by 3.0 mL distilled water. The glycans released by PNGase F were loaded on PGC cartridges and then washed with distilled water (3.0 mL) to remove buffer and salts. Glycans were eluted with 25% ACN in 0.1% TFA (3 × 1 mL). The fraction was collected and dried for further processing. The liquid phase was recovered and dried using a SpeedVac concentrator.

### Permethylation of glycans

Dried oligosaccharide sample was dissolved in an Eppendorf tube using 50 μL of DMSO. 100 μL of DMSO-NaOH slurry and 50 μL of methyl iodide were then added. Tubes were capped tightly and stirred for about 10 min at room temperature. The reaction was stopped by the addition of distilled water (0.5 mL). The permethylated oligosaccharides were extracted into chloroform (0.25 mL) by vortex mixing. The lower organic layer containing the permethylated oligosaccharides was washed with water (3 × 0.5 mL).

### Methylamidation of glycans

Dried glycans were dissolved in 25 μL of DMSO solution containing 1 M of methylamine hydrochloride and 0.5 M of *N*-methylmorpholine, followed by addition of 25 μL PyAOP (50 mM in DMSO) solution. The reaction mixture was vortexed and allowed to proceed at room temperature for 30 min. The glycan derivatives were purified according to a hydrophilic method described previously^32^. Briefly, cellulose particles were first washed with distilled water and then with solvent mixture of 1-butanol/ethanol/H_2_O (4:4:1, v/v/v). The reaction mixture solution was mixed with 5 mg cellulose particles in 1 mL of the organic solvent described above. After gentle shaking for 45 min, the cellulose particles were washed three times with 0.5 mL of the organic solvent. The cellulose particles were incubated with 0.3 mL of a mixture of ethanol/H_2_O (1:2 v/v) for 30 min. The liquid phase was recovered and dried using a SpeedVac concentrator. All samples were redissolved in distilled water prior to MS analysis.

### MALDI-MS analysis

The MALDI-MS spectra were acquired using 4800 MALDI-TOF/TOF (SCIEX, Concord, Canada) equipped with an Nd:YAG laser with 355 nm wavelength of <500 ps pulse and 200 Hz repetition rate. The spectrometer was operated in the positive reflectron mode. The spectra were accumulated by 1000 laser shots. The MS data were further processed using Dataexplorer 4.0. The samples were loaded onto MALDI target in 0.5 μL of water and mixed with 0.5 μL of freshly prepared DHB solution (10 mg/mL in 50% ACN) and allowed to dry in a gentle stream of air.

### LC-MS/MS analysis

LC-MS/MS experiments were performed using a TripleTOF 5600 System (SCIEX, Canada) and a NanoLC Ultra System (Eksigent, USA), equipped with a trap column (150 μm i.d. × 1 cm long; PGC, 5 μm; Proteomics Front, China) and a separation column (75 μm i.d. × 10 cm long; PGC, 5 μm; Proteomics Front, China). MS was operated in the positive-ion mode with a mass range of 500–3000 *m/z*, and MS/MS was acquired in the information dependent acquisition (IDA) mode with a mass range of 100–2000 *m/z*. The 20 most abundant precursor ions with charge numbers from 2 to 5 were scanned in the IDA mode. Each cycle consisted of a MS acquisition for 0.25 s and a total of 20 MS/MS scans for 2 s.

### Repeatability analysis

Repeatability of methylamidation conditions was investigated by multiple analyses of the same serum samples, including glycan releasing and purification. Using similar experiment design as for testing the repeatability of ethyl esterification conditions^38^, we repeated the entire procedure twice on consecutive days with freshly prepared reagents to establish day-to-day variability. For comparison, the native glycans from the same sample were also analysed using a TripleTOF 5600 System.

## Additional Information

**How to cite this article:** Wu, Z. *et al*. Characterization of *O*-acetylation in sialoglycans by MALDI-MS using a combination of methylamidation and permethylation. *Sci. Rep.* **7**, 46206; doi: 10.1038/srep46206 (2017).

**Publisher's note:** Springer Nature remains neutral with regard to jurisdictional claims in published maps and institutional affiliations.

## Supplementary Material

Supplementary Information

## References

[b1] Dwek R. A. Glycobiology: more functions for oligosaccharides. Science 269, 1234–1235 (1995). 7652569 10.1126/science.7652569

[b2] Varki A. Glycan-based interactions involving vertebrate sialic-acid-recognizing proteins. Nature 446, 1023–1029 (2007). 17460663 10.1038/nature05816

[b3] Sun S. . Comprehensive analysis of protein glycosylation by solid-phase extraction of N-linked glycans and glycosite-containing peptides. Nat. Biotechnol. 34, 84–88 (2016). 26571101 10.1038/nbt.3403 PMC4872599

[b4] Bondt A. . Longitudinal monitoring of immunoglobulin A glycosylation during pregnancy by simultaneous MALDI-FTICR-MS analysis of N- and O-glycopeptides. Sci. Rep. 6, 27955 (2016). 27302155 10.1038/srep27955 PMC4908400

[b5] Varki A. Colloquium paper: uniquely human evolution of sialic acid genetics and biology. Proc. Natl. Acad. Sci. USA 107 Suppl 2, 8939–8946 (2010). 20445087 10.1073/pnas.0914634107 PMC3024026

[b6] Varki A. Diversity in the sialic acids. Glycobiology 2, 25–40 (1992). 1550987 10.1093/glycob/2.1.25 PMC7108601

[b7] Klein A. & Roussel P. O-acetylation of sialic acids. Biochimie 80, 49–57 (1998). 9587662 10.1016/s0300-9084(98)80056-4

[b8] Schauer R. Achievements and challenges of sialic acid research. Glycoconj. J. 17, 485–499 (2000). 11421344 10.1023/A:1011062223612 PMC7087979

[b9] Campbell F. . Racial variation in the O-acetylation phenotype of human colonic mucosa. J. Pathol. 174, 169–174 (1994). 7823249 10.1002/path.1711740305

[b10] Diaz S., Higa H. H., Hayes B. K. & Varki A. O-acetylation and de-O-acetylation of sialic acids. 7- and 9-o-acetylation of alpha 2,6-linked sialic acids on endogenous N-linked glycans in rat liver Golgi vesicles. J. Biol. Chem. 264, 19416–19426 (1989). 2808433

[b11] Diaz S. L. . Sensitive and specific detection of the non-human sialic Acid N-glycolylneuraminic acid in human tissues and biotherapeutic products. PLoS. One. 4, e4241 (2009). 19156207 10.1371/journal.pone.0004241 PMC2626223

[b12] Higa H. H., Manzi A. & Varki A. O-acetylation and de-O-acetylation of sialic acids. Purification, characterization, and properties of a glycosylated rat liver esterase specific for 9-O-acetylated sialic acids. J. Biol. Chem. 264, 19435–19442 (1989). 2808434

[b13] Shen Y. . Characterization of the sialate-7 (9)-O-acetyltransferase from the microsomes of human colonic mucosa. Biol. Chem. 383, 307–317 (2002). 11934269 10.1515/BC.2002.033

[b14] Shen Y. . O-acetylation and de-O-acetylation of sialic acids in human colorectal carcinoma. Eur. J. Biochem. 271, 281–290 (2004). 14717696 10.1046/j.1432-1033.2003.03927.x

[b15] Shi W. X., Chammas R. & Varki A. Regulation of sialic acid 9-O-acetylation during the growth and differentiation of murine erythroleukemia cells. J. Biol. Chem. 271, 31517–31525 (1996). 8940167 10.1074/jbc.271.49.31517

[b16] Haverkamp J. . High-resolution 1H-NMR spectroscopy of free and glycosidically linked O-acetylated sialic acids. Eur. J. Biochem. 122, 305–311 (1982). 7060578 10.1111/j.1432-1033.1982.tb05881.x

[b17] Surolia I. . Functionally defective germline variants of sialic acid acetylesterase in autoimmunity. Nature 466, 243–247 (2010). 20555325 10.1038/nature09115 PMC2900412

[b18] Samraj A. N. . A red meat-derived glycan promotes inflammation and cancer progression. Proc. Natl. Acad. Sci. USA 112, 542–547 (2015). 25548184 10.1073/pnas.1417508112 PMC4299224

[b19] de H. N. . Linkage-Specific Sialic Acid Derivatization for MALDI-TOF-MS Profiling of IgG Glycopeptides. Anal. Chem. 87, 8284–8291 (2015). 26191964 10.1021/acs.analchem.5b02426

[b20] Hua S. . Isomer-specific chromatographic profiling yields highly sensitive and specific potential N-glycan biomarkers for epithelial ovarian cancer. J. Chromatogr. A 1279, 58–67 (2013). 23380366 10.1016/j.chroma.2012.12.079 PMC5628020

[b21] Lebrilla C. B. & An H. J. The prospects of glycan biomarkers for the diagnosis of diseases. Mol. Biosyst. 5, 17–20 (2009). 19081926 10.1039/b811781k

[b22] Harvey D. J. . MALDI-MS/MS with traveling wave ion mobility for the structural analysis of N-linked glycans. J. Am. Soc. Mass Spectrom. 23, 1955–1966 (2012). 22993039 10.1007/s13361-012-0425-8

[b23] Pang P. C. . Human sperm binding is mediated by the sialyl-Lewis(x) oligosaccharide on the zona pellucida. Science 333, 1761–1764 (2011). 21852454 10.1126/science.1207438

[b24] Dell A. & Morris H. R. Glycoprotein structure determination by mass spectrometry. Science 291, 2351–2356 (2001). 11269315 10.1126/science.1058890

[b25] Adamczyk B. . Pregnancy-associated changes of IgG and serum N-glycosylation in camel (camelus dromedarius). J. Proteome. Res. 15, 3255–3265 (2016). 27428249 10.1021/acs.jproteome.6b00439

[b26] Liu X. . O-acetylation of sialic acids in N-glycans of Atlantic salmon (Salmo salar) serum is altered by handling stress. Proteomic. 8, 2849–2857 (2008). 10.1002/pmic.200701093 18655054

[b27] Jayo R. G., Li J. & Chen D. D. Capillary electrophoresis mass spectrometry for the characterization of O-acetylated N-glycans from fish serum. Anal. Chem. 84, 8756–8762 (2012). 22971167 10.1021/ac301889k

[b28] Harvey D. J. Analysis of carbohydrates and glycoconjugates by matrix-assisted laser desorption/ionization mass spectrometry: An update for 2011–2012. Mass Spectrom. Rev(2015). 10.1002/mas.21411 PMC7168572 24863367

[b29] Alley W. R. Jr., Mann B. F. & Novotny M. V. High-sensitivity analytical approaches for the structural characterization of glycoproteins. Chem. Rev. 113, 2668–2732 (2013). 23531120 10.1021/cr3003714 PMC3992972

[b30] Everest-Dass A. V. . N-Glycan MALDI Imaging Mass Spectrometry on Formalin-Fixed Paraffin-Embedded Tissue Enables the Delineation of Ovarian Cancer Tissues. Mol. Cell Proteomics 15, 3003–3016 (2016). 27412689 10.1074/mcp.M116.059816 PMC5013313

[b31] Holst S. . Linkage-Specific *in Situ* Sialic Acid Derivatization for N-Glycan Mass Spectrometry Imaging of Formalin-Fixed Paraffin-Embedded Tissues. Anal. Chem. 88, 5904–5913 (2016). 27145236 10.1021/acs.analchem.6b00819

[b32] Liu X., Qiu H., Lee R. K., Chen W. & Li J. Methylamidation for sialoglycomics by MALDI-MS: a facile derivatization strategy for both alpha2,3- and alpha2,6-linked sialic acids. Anal. Chem. 82, 8300–8306 (2010). 20831242 10.1021/ac101831t

[b33] Nishikaze T., Kawabata S. & Tanaka K. In-depth structural characterization of N-linked glycopeptides using complete derivatization for carboxyl groups followed by positive- and negative-ion tandem mass spectrometry. Anal. Chem. 86, 5360–5369 (2014). 24773001 10.1021/ac500340t

[b34] Zhang Q. . Methylamidation for isomeric profiling of sialylated glycans by nanoLC-MS. Anal. Chem. 86, 7913–7919 (2014). 25022802 10.1021/ac501844b

[b35] Zhou H., Warren P. G., Froehlich J. W. & Lee R. S. Dual modifications strategy to quantify neutral and sialylated N-glycans simultaneously by MALDI-MS. Anal. Chem. 86, 6277–6284 (2014). 24766348 10.1021/ac500298a PMC4082391

[b36] Wheeler S. F., Domann P. & Harvey D. J. Derivatization of sialic acids for stabilization in matrix-assisted laser desorption/ionization mass spectrometry and concomitant differentiation of alpha(2 –>3)- and alpha(2 –>6)-isomers. Rapid Commun. Mass Spectrom. 23, 303–312 (2009). 19089860 10.1002/rcm.3867

[b37] Bladergroen M. R. . Automation of High-Throughput Mass Spectrometry-Based Plasma N-Glycome Analysis with Linkage-Specific Sialic Acid Esterification. J. Proteome. Res. 14, 4080–4086 (2015). 26179816 10.1021/acs.jproteome.5b00538

[b38] Reiding K. R., Blank D., Kuijper D. M., Deelder A. M. & Wuhrer M. High-throughput profiling of protein N-glycosylation by MALDI-TOF-MS employing linkage-specific sialic acid esterification. Anal. Chem. 86, 5784–5793 (2014). 24831253 10.1021/ac500335t

[b39] Cao L. . Global food supply. China’s aquaculture and the world’s wild fisheries. Science 347, 133–135 (2015). 25574011 10.1126/science.1260149

[b40] Inoue S., Iwasaki M., Ishii K., Kitajima K. & Inoue Y. Isolation and structures of glycoprotein-derived free sialooligosaccharides from the unfertilized eggs of Tribolodon hakonensis, a dace. Intracellular accumulation of a novel class of biantennary disialooligosaccharides. J. Biol. Chem. 264, 18520–18526 (1989). 2808387

[b41] Ishii K. . Free sialooligosaccharides found in the unfertilized eggs of a freshwater trout, Plecoglossus altivelis. A large storage pool of complex-type bi-, tri-, and tetraantennary sialooligosaccharides. J. Biol. Chem. 264, 1623–1630 (1989). 2912977

[b42] Taguchi T. . Structural studies of a novel type of tetraantennary sialoglycan unit in a carbohydrate-rich glycopeptide isolated from the fertilized eggs of Indian Medaka fish, Oryzias melastigma. J. Biol. Chem. 268, 2353–2362 (1993). 8381405

[b43] Taguchi T. . Structural studies of a novel type of pentaantennary large glycan unit in the fertilization-associated carbohydrate-rich glycopeptide isolated from the fertilized eggs of Oryzias latipes. J. Biol. Chem. 269, 8762–8771 (1994). 8132608

[b44] Taguchi T. . A precise structural analysis of a fertilization-associated carbohydrate-rich glycopeptide isolated from the fertilized eggs of euryhaline killi fish (Fundulus heteroclitus). Novel penta-antennary N-glycan chains with a bisecting N-acetylglucosaminyl residue. Glycobiology 5, 611–624 (1995). 8563149 10.1093/glycob/5.6.611

