# Supplementary material for: Characterization of O-acetylation in sialoglycans by MALDI-MS using a combination of methylamidation and permethylation
Source: Sci Rep. 2017 Apr 7;7:46206. doi: 10.1038/srep46206 (PMC5384204; doi:10.1038/srep46206)
Supplement: Supplementary Information [file srep46206-s1.pdf]

Supporting Information

Characterization of *O*-acetylation in sialoglycans by MALDI-MS using a combination of methylamidation and permethylation

Zhaoguan Wu[1], Henghui Li[2], Qiwei Zhang[1], Xin Liu[2], Qi Zheng[1]*, Jianjun Li[3]*

[1] Key Laboratory of Optoelectronic Chemical Materials and Devices of Ministry of Education, Institute for Interdisciplinary Research, School of Chemical and Environmental Engineering, Jianghan University, Wuhan, China 430056

[2] College of Life Science and Technology, Huazhong University of Science and Technology, Wuhan, China 430074

[3] Human Health Therapeutics, National Research Council Canada, 100 Sussex Drive, Ottawa, ON, Canada K1A 0R6

* Corresponding authors:

Dr. Qi Zheng, School of Chemical and Environmental Engineering, Jianghan University, Wuhan, China 430056, E-mail: zq_1101@sina.com

Dr. Jianjun Li, Human Health Therapeutics, National Research Council Canada, 100 Sussex Drive, Ottawa, ON, Canada K1A 0R6, E-mail: Jianjun.Li@nrc-cnrc.gc.ca

Table of Contents

Figure S1.	LC-MS/MS analysis of methylamidated N-glycans isolated from a representative serum sample of crucian carp (Crucian-1).
Figure S2.	MALDI-MS analysis of permethylated and methylamidated N-glycans isolated from three serum sample replicates of crucian carp.
Table S1.	Detected ions and their corresponding compositions of N-glycans from crucian carp serum samples.
Figure S3.	LC-MS/MS analysis of methylamidated N-glycans isolated from a representative serum sample of common carp (Common-1).
Figure S4.	MALDI-MS analysis of permethylated and methylamidated N-glycans isolated from three serum sample replicates of common carp.
Table S2.	Detected ions and their corresponding compositions of N-glycans from common carp serum samples.
Figure S5.	LC-MS/MS analysis of methylamidated N-glycans isolated from a representative serum sample of grass carp (Grass-1).
Figure S6.	MALDI-MS analysis of permethylated and methylamidated N-glycans isolated from three serum sample replicates of grass carp.
Table S3.	Detected ions and their corresponding compositions of N-glycans from grass carp serum samples.
Figure S7.	LC-MS/MS analysis of methylamidated N-glycans isolated from a representative serum sample of silver carp (Silver-1).
Figure S8.	MALDI-MS analysis of permethylated and methylamidated N-glycans isolated from three serum sample replicates of silver carp.
Table S4.	Detected ions and their corresponding compositions of N-glycans from silver carp serum samples.

Figure S9. LC-MS/MS analysis of methylamidated N-glycans isolated from a representative serum sample of bream carp (Bream-1).

Figure S10. MALDI-MS analysis of permethylated and methylamidated N-glycans isolated from three serum sample replicates of bream carp.

Table S5. Detected ions and their corresponding compositions of N-glycans from bream carp serum samples.

Figure S11. LC-MS/MS analysis of methylamidated N-glycans isolated from a representative serum sample of bighead carp (Bighead-1).

Figure S12. MALDI-MS analysis of permethylated and methylamidated N-glycans isolated from three serum sample replicates of bighead carp.

Table S6. Detected ions and their corresponding compositions of N-glycans from bighead carp serum samples.

Figure S13. LC-MS/MS analysis of methylamidated N-glycans isolated from a representative serum sample of black carp (Black-1).

Figure S14. MALDI-MS analysis of permethylated and methylamidated N-glycans isolated from three serum sample replicates of black carp.

Table S7. Detected ions and their corresponding compositions of N-glycans from black carp serum samples.

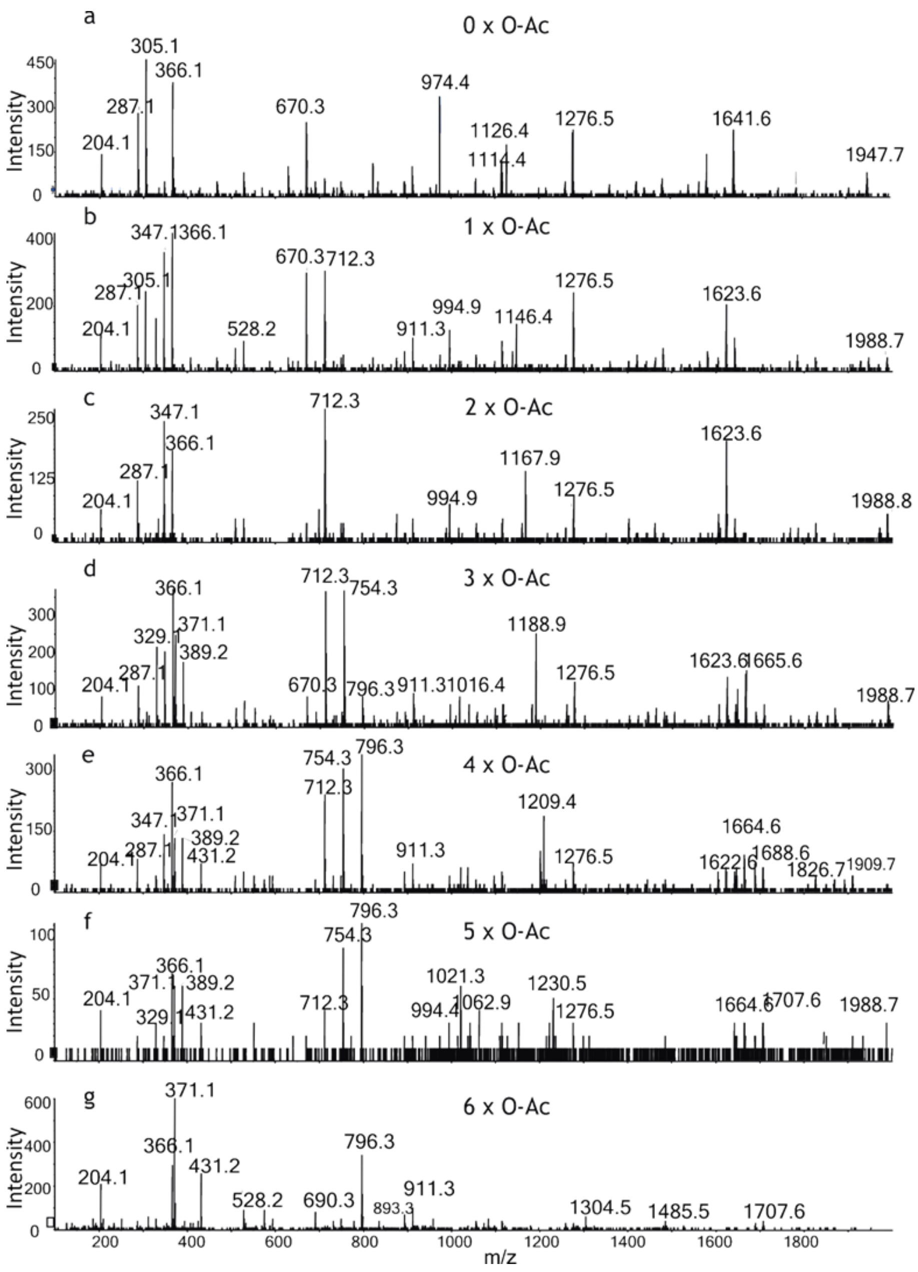

Figure S1. LC-MS/MS analysis of methylamidated N-glycans isolated from a representative serum sample of crucian carp (Crucian-1). (a) MS/MS spectrum of $[M+2H]^{2+}$ at *m/z* 1125.3 ($Neu5NAc_2Hex_5HexNAc_4$); (b) MS/MS spectrum of $[M+2H]^{2+}$ at *m/z* 1146.4 ($OAc_1Neu5NAc_2Hex_5HexNAc_4$); (c) MS/MS spectrum of $[M+2H]^{2+}$ at *m/z* 1167.9 ($OAc_2Neu5NAc_2Hex_5HexNAc_4$); (d) MS/MS spectrum of $[M+2H]^{2+}$ at *m/z* 1188.9 ($OAc_3Neu5NAc_2Hex_5HexNAc_4$); (e) MS/MS spectrum of $[M+2H]^{2+}$ at *m/z* 1209.4 ($OAc_4Neu5NAc_2Hex_5HexNAc_4$); (f) MS/MS spectrum of $[M+2H]^{2+}$ at *m/z* 1230.4 ($OAc_5Neu5NAc_2Hex_5HexNAc_4$); (g) MS/MS spectrum of $[M+3H]^{3+}$ at *m/z* 834.6 ($OAc_6Neu5NAc_2Hex_5HexNAc_4$).

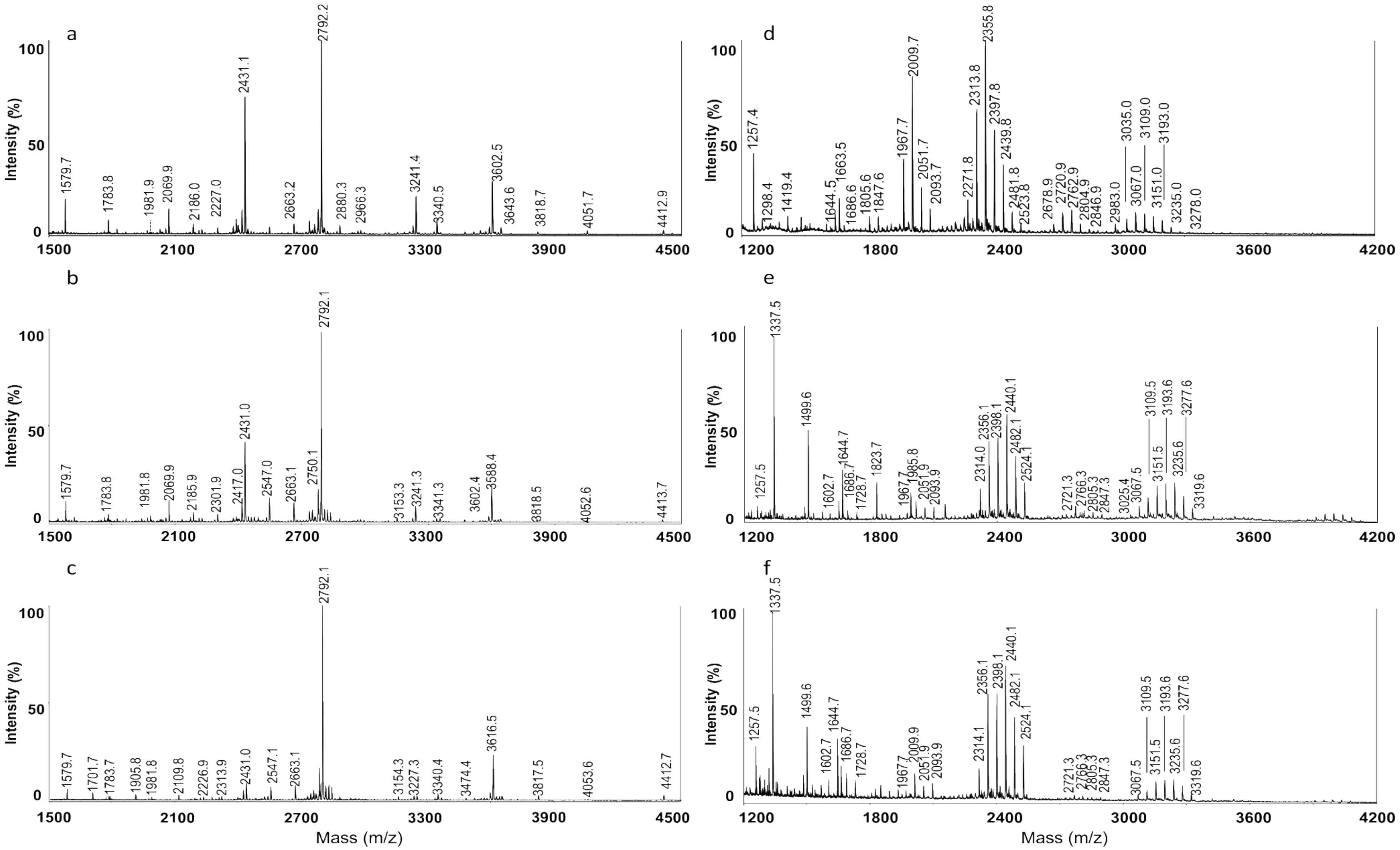

Figure S2. MALDI-MS analysis of N-glycans isolated from three serum sample replicates of crucian carp, labelled as Crucian-1, Crucian-2, Crucian-3: (a) permethylated glycans from Crucian-1 (see also Figure 1a); (b) permethylated glycans from Crucian-2; (c) permethylated glycans from Crucian-3; (d) methylamidated glycans from Crucian-1 (see also Figure 1b); (e) methylamidated glycans from Crucian-2; (f) methylamidated glycans from Crucian-3. The ions at *m/z* 1337.5 and *m/z* 1499.6 appear to be associated with hexose polymers, *i.e.* $[M+Na]^+$ of Hex_8 and Hex_9, respectively. These background ions possibly originated from the cellulose SPE.

Table S1. Detected ions and their corresponding compositions of N-glycans from crucian carp serum samples

**Proposed composition**	**Permethylation**		**Methylamidation**	
	Theoretical* $[M+Na]^+$ (*m/z*)	Detected $[M+Na]^+$ (*m/z*)	Theoretical $[M+Na]^+$ (*m/z*)	Detected $[M+Na]^+$ (*m/z*)
Hex$_5$HexNAc$_2$	1579.8	1579.7	1257.4	1257.4
Hex$_6$HexNAc$_2$	1783.9	1783.8	1419.5	1419.4
Neu5NAc$_1$Hex$_4$HexNAc$_3$	1982.0	1981.9	1602.6	1602.5
OAc$_1$Neu5NAc$_1$Hex$_4$HexNAc$_3$			1644.6	1644.5
OAc$_2$Neu5NAc$_1$Hex$_4$HexNAc$_3$			1686.6	1686.6
OAc$_3$Neu5NAc$_1$Hex$_4$HexNAc$_3$			1728.6	1782.6
Hex$_5$HexNAc$_4$	2070.0	2069.9	1663.6	1663.5
Neu5NAc$_1$Hex$_4$HexNAc$_4$	2227.1	2227.0	1805.7	1805.6
OAc$_1$Neu5NAc$_1$Hex$_4$HexNAc$_4$			1847.7	1847.6
OAc$_2$Neu5NAc$_1$Hex$_4$HexNAc$_4$			1889.7	1899.6
OAc$_3$Neu5NAc$_1$Hex$_4$HexNAc$_4$			1931.7	1931.6
Neu5NAc$_1$Hex$_5$HexNAc$_4$	2431.2	2431.1	1967.7	1967.7
OAc$_1$Neu5NAc$_1$Hex$_5$HexNAc$_4$			2009.7	2009.7
OAc$_2$Neu5NAc$_1$Hex$_5$HexNAc$_4$			2051.7	2051.7
OAc$_3$Neu5NAc$_1$Hex$_5$HexNAc$_4$			2093.7	2093.7
Neu5NAc$_2$Hex$_5$HexNAc$_4$	2792.4	2792.2	2271.8	2271.8
OAc$_1$Neu5NAc$_2$Hex$_5$HexNAc$_4$			2313.9	2313.8
OAc$_2$Neu5NAc$_2$Hex$_5$HexNAc$_4$			2355.9	2355.8
OAc$_3$Neu5NAc$_2$Hex$_5$HexNAc$_4$			2397.9	2397.8
OAc$_4$Neu5NAc$_2$Hex$_5$HexNAc$_4$			2439.9	2439.8
OAc$_5$Neu5NAc$_2$Hex$_5$HexNAc$_4$			2481.9	2481.8
OAc$_6$Neu5NAc$_2$Hex$_5$HexNAc$_4$			2523.9	2523.8
Neu5NAc$_1$Hex$_6$HexNAc$_5$	2880.4	2880.3	2332.8	2332.7
Neu5NAc$_2$Hex$_6$HexNAc$_5$	3241.6	3241.4	2637.0	2336.8
OAc$_1$Neu5NAc$_2$Hex$_6$HexNAc$_5$			2679.0	2678.9
OAc$_2$Neu5NAc$_2$Hex$_6$HexNAc$_5$			2721.0	2720.9
OAc$_3$Neu5NAc$_2$Hex$_6$HexNAc$_5$			2763.0	2762.9
OAc$_4$Neu5NAc$_2$Hex$_6$HexNAc$_5$			2805.0	2804.9
OAc$_5$Neu5NAc$_2$Hex$_6$HexNAc$_5$			2847.0	2864.9
OAc$_6$Neu5NAc$_2$Hex$_6$HexNAc$_5$			2889.0	2888.9
Neu5NAc$_3$Hex$_6$HexNAc$_5$	3602.8	3602.5	2941.1	2940.9
OAc$_1$Neu5NAc$_3$Hex$_6$HexNAc$_5$			2983.1	2983.0

$OAc_2Neu5NAc_3Hex_6HexNAc_5$			3025.1	3025.0
$OAc_3Neu5NAc_3Hex_6HexNAc_5$			3067.1	3067.0
$OAc_4Neu5NAc_3Hex_6HexNAc_5$			3109.1	3109.0
$OAc_5Neu5NAc_3Hex_6HexNAc_5$			3151.1	3151.0
$OAc_6Neu5NAc_3Hex_6HexNAc_5$			3193.2	3193.0
$OAc_7Neu5NAc_3Hex_6HexNAc_5$			3235.2	3235.0
$OAc_8Neu5NAc_3Hex_6HexNAc_5$			3277.2	3278.0
$OAc_9Neu5NAc_3Hex_6HexNAc_5$			3319.2	3319.1
$Neu5NAc_3Hex_7HexNAc_6$	4052.0	4051.7	3306.2	ND**
$OAc_3Neu5NAc_3Hex_7HexNAc_6$			3432.3	3433.1
$OAc_4Neu5NAc_3Hex_7HexNAc_6$			3747.3	3475.3
$OAc_5Neu5NAc_3Hex_7HexNAc_6$			3616.3	3517.1
$OAc_6Neu5NAc_3Hex_7HexNAc_6$			3558.3	3559.2
$Neu5NAc_4Hex_7HexNAc_6$	4413.2	4412.9	3610.4	ND
$OAc_4Neu5NAc_4Hex_7HexNAc_6$			3778.4	3779.1
$OAc_5Neu5NAc_4Hex_7HexNAc_6$			3820.4	3821.1
$OAc_6Neu5NAc_4Hex_7HexNAc_6$			3862.4	3863.2
$OAc_7Neu5NAc_4Hex_7HexNAc_6$			3904.4	3905.1
$OAc_8Neu5NAc_4Hex_7HexNAc_6$			3946.4	3947.1
$OAc_9Neu5NAc_4Hex_7HexNAc_6$			3988.4	3989.1
$OAc_{10}Neu5NAc_4Hex_7HexNAc_6$			4030.5	4031.3

* Monoisotopic mass units were used for calculation of molecular mass based on proposed composition.
** Not detected.

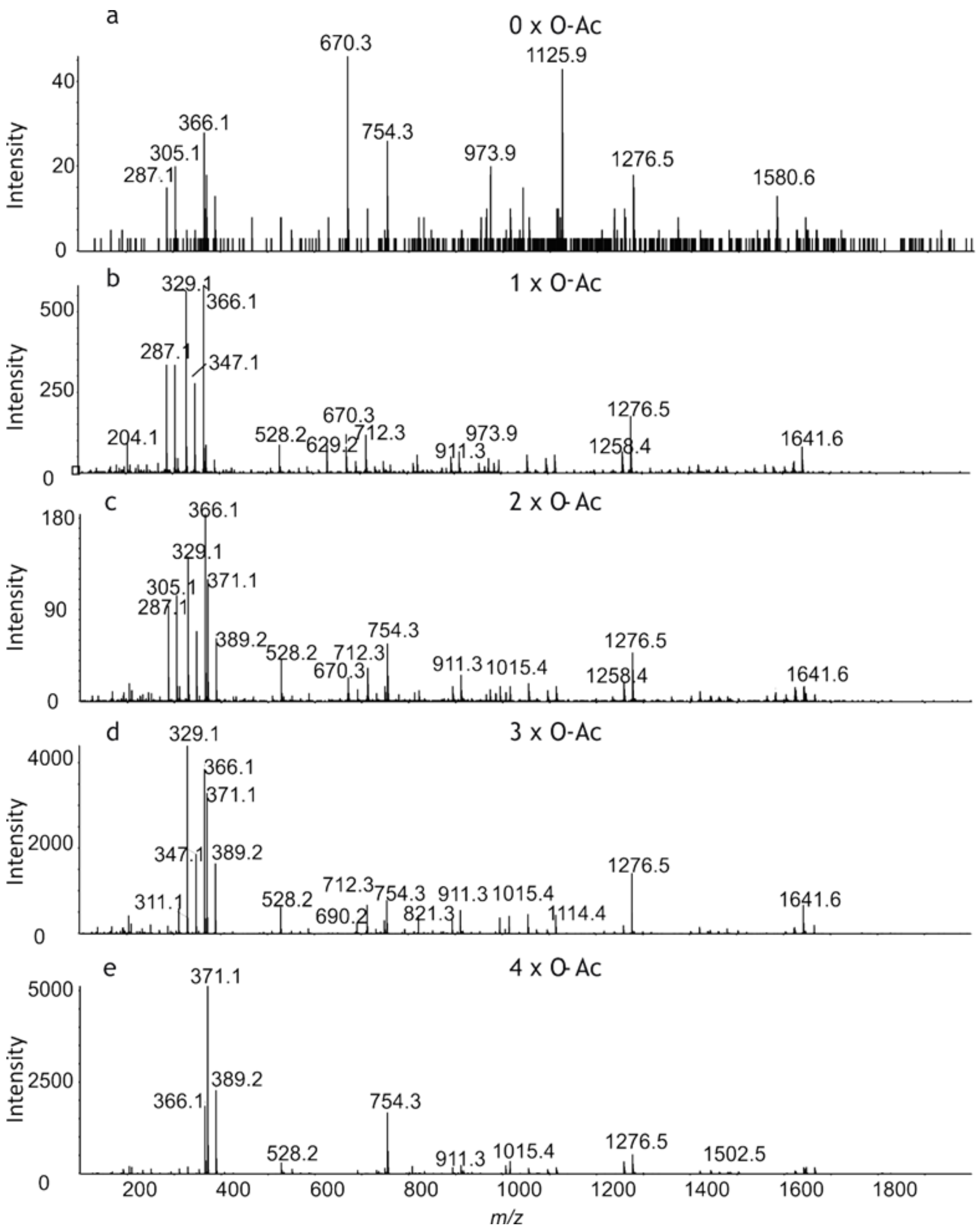

Figure S3. LC-MS/MS analysis of methylamidated N-glycans isolated from a representative serum sample of common carp (Common-1). (a) MS/MS spectrum of $[M+2H]^{2+}$ at *m/z* 1125.4 ($Neu5NAc_2Hex_5HexNAc_4$); (b) MS/MS spectrum of $[M+3H]^{3+}$ at *m/z* 764.3 ($OAc_1Neu5NAc_2Hex_5HexNAc_4$); (c) MS/MS spectrum of $[M+3H]^{3+}$ at *m/z* 778.6 ($OAc_2Neu5NAc_2Hex_5HexNAc_4$); (d) MS/MS spectrum of $[M+3H]^{3+}$ at *m/z* 792.6 ($OAc_3Neu5NAc_2Hex_5HexNAc_4$); (e) MS/MS spectrum of $[M+3H]^{3+}$ at *m/z* 806.6 ($OAc_4Neu5NAc_2Hex_5HexNAc_4$).

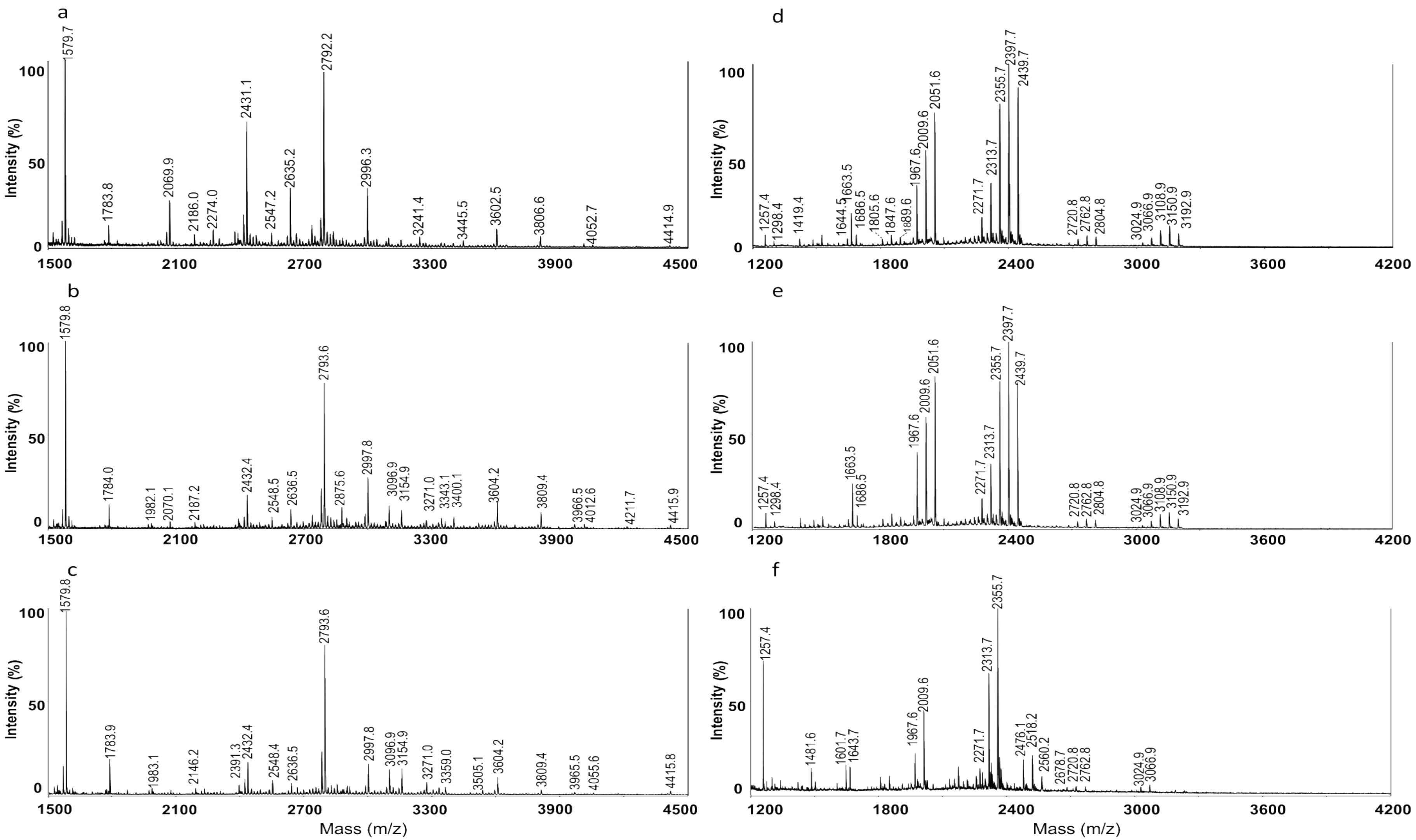

Figure S4. MALDI-MS analysis of N-glycans isolated from three serum sample replicates of common carp, labelled as Common-1, Common-2, Common-3: (a) permethylated glycans from Common-1 (see also Figure 3a); (b) permethylated glycans from Common-2; (c) permethylated glycans from Common-3; (d) methylamidated glycans from Common-1 (see also Figure 3b); (e) methylamidated glycans from Common-2; (f) methylamidated glycans from Common-3.

Table S2. Detected ions and their corresponding compositions of N-glycans from common carp serum samples

**Proposed composition**	**Permethylation**		**Methylamidation**	
	Theoretical* [M+Na]$^+$ (*m/z*)	Detected [M+Na]$^+$ (*m/z*)	Theoretical [M+Na]$^+$ (*m/z*)	Detected [M+Na]$^+$ (*m/z*)
$Hex_5HexNAc_2$	1579.8	1579.7	1257.4	1257.4
$Hex_6HexNAc_2$	1783.9	1783.8	1419.5	1419.4
$Neu5NAc_1Hex_4HexNAc_3$	1982.0	1981.9	1602.6	1602.5
$OAc_1Neu5NAc_1Hex_4HexNAc_3$			1644.6	1644.5
$OAc_2Neu5NAc_1Hex_4HexNAc_3$			1686.6	1686.5
$Hex_5HexNAc_4$	2070.0	2069.9	1663.6	1663.5
$Neu5NAc_1Hex_4HexNAc_4$	2227.1	2227.0	1805.7	1805.6
$OAc_1Neu5NAc_1Hex_4HexNAc_4$			1847.7	1847.6
$OAc_2Neu5NAc_1Hex_4HexNAc_4$			1889.7	1899.6
$OAc_3Neu5NAc_1Hex_4HexNAc_4$			1931.7	1931.6
$Hex_6HexNAc_4$	2274.1	2274.0	1825.6	ND**
$Neu5NAc_1Hex_5HexNAc_4$	2431.2	2431.1	1967.7	1967.6
$OAc_1Neu5NAc_1Hex_5HexNAc_4$			2009.7	2009.6
$OAc_2Neu5NAc_1Hex_5HexNAc_4$			2051.7	2051.6
$OAc_3Neu5NAc_1Hex_5HexNAc_4$			2093.7	2093.6
$Neu5NAc_2Hex_5HexNAc_4$	2792.4	2792.2	2271.8	2271.7
$OAc_1Neu5NAc_2Hex_5HexNAc_4$			2313.9	2313.7
$OAc_2Neu5NAc_2Hex_5HexNAc_4$			2355.9	2355.7
$OAc_3Neu5NAc_2Hex_5HexNAc_4$			2397.9	2397.7
$OAc_4Neu5NAc_2Hex_5HexNAc_4$			2439.9	2439.7
$Neu5NAc_2Hex_6HexNAc_5$	3241.6	3241.4	2637.0	2336.8
$OAc_1Neu5NAc_2Hex_6HexNAc_5$			2679.0	2678.8
$OAc_2Neu5NAc_2Hex_6HexNAc_5$			2721.0	2720.8
$OAc_3Neu5NAc_2Hex_6HexNAc_5$			2763.0	2762.8
$OAc_4Neu5NAc_2Hex_6HexNAc_5$			2805.0	2804.8
$Neu5NAc_2Hex_7HexNAc_5$	3445.7	3445.5	2799.0	ND
$Neu5NAc_3Hex_6HexNAc_5$	3602.8	3602.5	2941.1	ND
$OAc_1Neu5NAc_3Hex_6HexNAc_5$			2983.1	2982.9
$OAc_2Neu5NAc_3Hex_6HexNAc_5$			3025.1	3024.9
$OAc_3Neu5NAc_3Hex_6HexNAc_5$			3067.1	3066.9
$OAc_4Neu5NAc_3Hex_6HexNAc_5$			3109.1	3108.9
$OAc_5Neu5NAc_3Hex_6HexNAc_5$			3151.1	3150.9
$OAc_6Neu5NAc_3Hex_6HexNAc_5$			3193.2	3192.9
$Neu5NAc_3Hex_7HexNAc_6$	4052.0	4052.7	3306.2	ND

* Monoisotopic mass units were used for calculation of molecular mass based on proposed composition.
** Not detected.

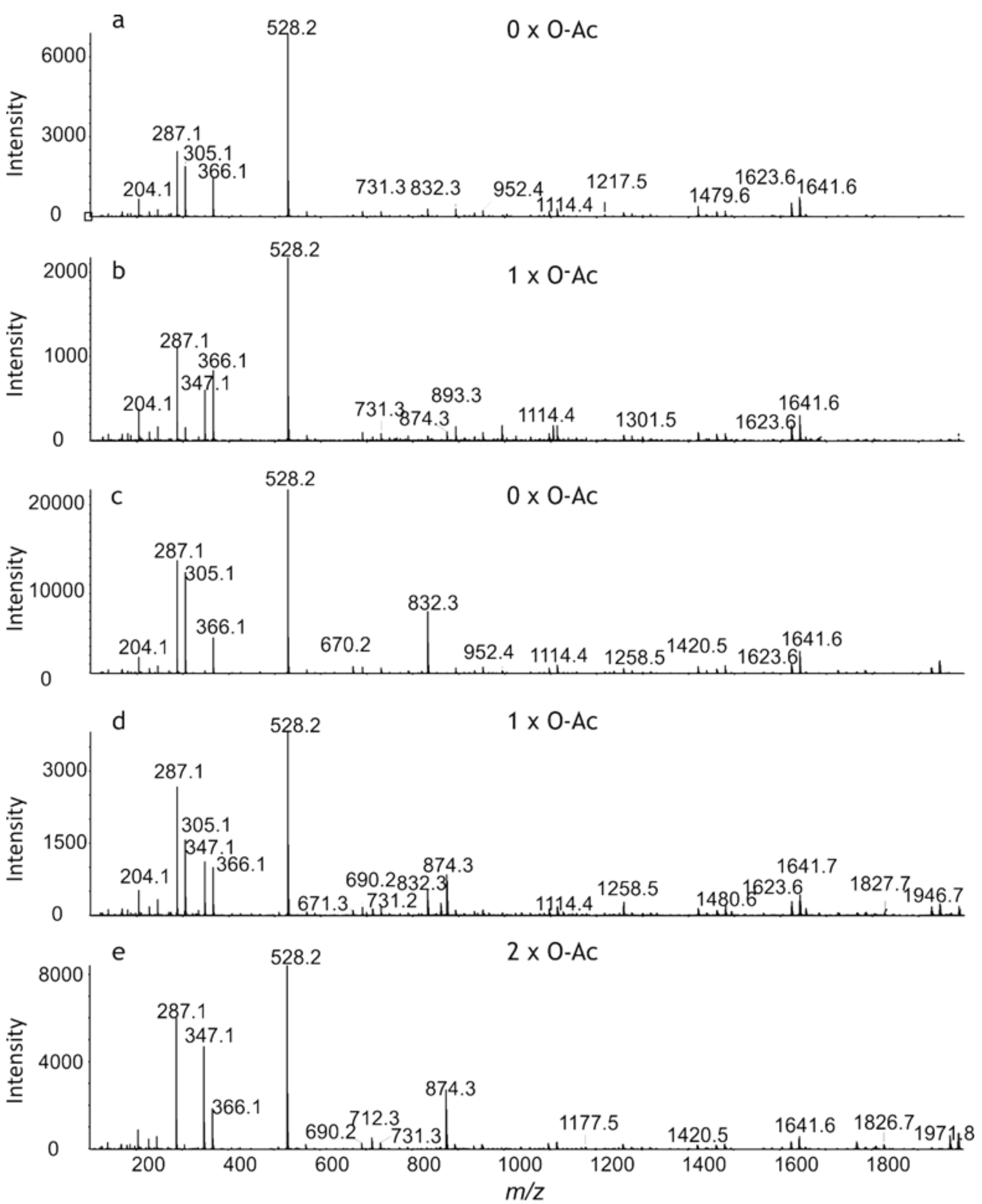

Figure S5. LC-MS/MS analysis of methylamidated N-glycans isolated from a representative serum sample of grass carp (Grass-1). (a) MS/MS spectrum of $[M+3H]^{3+}$ at *m/z* 825.0 ($Neu5NAc_1Hex_7HexNAc_5$); (b) MS/MS spectrum of $[M+3H]^{3+}$ at *m/z* 839.0 ($OAc_1Neu5NAc_1Hex_7HexNAc_5$); (c) MS/MS spectrum of $[M+3H]^{3+}$ at *m/z* 926.4 ($Neu5NAc_2Hex_7HexNAc_5$); (d) MS/MS spectrum of $[M+3H]^{3+}$ at *m/z* 940.4 ($OAc_1Neu5NAc_2Hex_7HexNAc_5$); (e) MS/MS spectrum of $[M+3H]^{3+}$ at *m/z* 954.4 ($OAc_2Neu5NAc_2Hex_7HexNAc_5$).

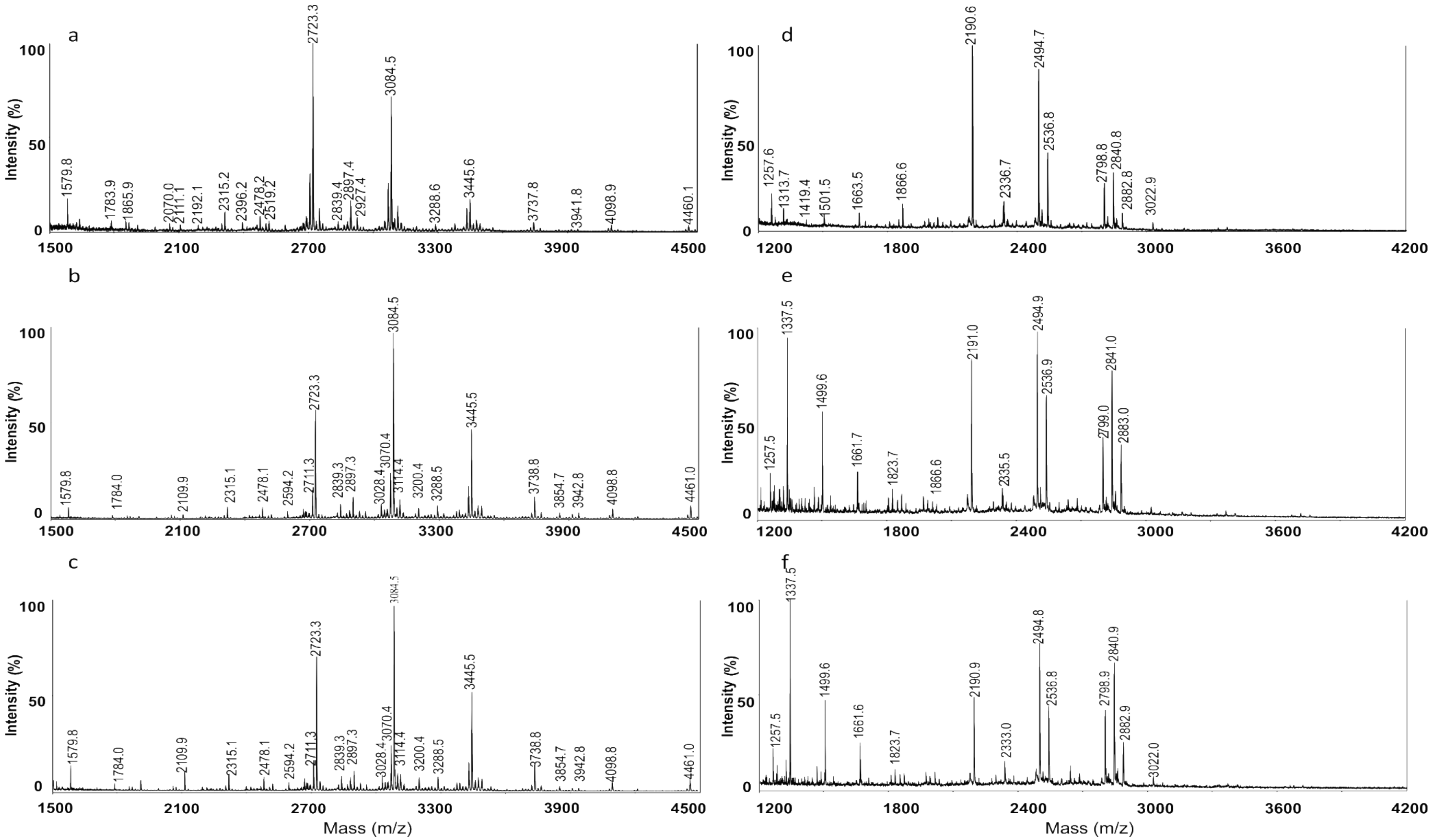

Figure S6. MALDI-MS analysis of N-glycans isolated from three serum sample replicates of grass carp, labelled as Grass-1, Grass-2, Grass-3: (a) permethylated glycans from Grass-1 (see also Figure 4a); (b) permethylated glycans from Grass-2; (c) permethylated glycans from Grass-3; (d) methylamidated glycans from Grass-1 (see also Figure 4b); (e) methylamidated glycans from Grass-2; (f) methylamidated glycans from Grass-3. The ions at *m/z* 1337.5, *m/z* 1499.6 and *m/z* 1661.7 appear to be associated with hexose polymers, *i.e.* $[M+Na]^+$ of Hex_8, Hex_9 and Hex_{10}, respectively. These background ions possibly originated from the cellulose SPE.

Table S3. Detected ions and their corresponding compositions of N-glycans from grass carp serum samples

Proposed composition	Permethylation		Methylamidation	
	Theoretical* $[M+Na]^+$ (*m/z*)	Detected $[M+Na]^+$ (*m/z*)	Theoretical $[M+Na]^+$ (*m/z*)	Detected $[M+Na]^+$ (*m/z*)
$Hex_5HexNAc_2$	1579.8	1579.7	1257.4	1257.4
$Hex_6HexNAc_2$	1783.9	1783.9	1419.5	1419.4
$Hex_4HexNAc_4$	1865.9	1865.9	1501.5	1501.5
$Hex_7HexNAc_2$	1988.0	1987.9	1581.5	ND**
$Hex_5HexNAc_4$	2070.0	2070.0	1663.6	1663.5
$Hex_8HexNAc_2$	2192.1	2192.1	1743.6	1743.5
$Hex_6HexNAc_4$	2274.1	2274.1	1825.6	1825.6
$Hex_5HexNAc_5$	2315.2	2315.2	1866.7	1866.6
$Hex_9HexNAc_2$	2396.2	2396.2	1905.6	1905.5
$Hex_7HexNAc_4$	2478.2	2478.2	1987.7	1987.6
$Hex_6HexNAc_5$	2519.3	2519.2	2028.7	2028.6
$Hex_7HexNAc_5$	2723.4	2723.3	2190.8	2190.6
$Neu5NAc_1Hex_7HexNAc_4$	2839.4	2839.4	2291.8	2291.7
$Fuc_1Hex_6HexNAc_5$	2897.5	2897.4	2336.8	2336.7
$Hex_8HexNAc_5$	2927.5	2927.4	2352.8	2352.7
$Neu5NAc_1Hex_7HexNAc_5$	3084.5	3084.5	2494.9	2494.7
$OAc_1Neu5NAc_1Hex_7HexNAc_5$			2536.9	2536.8
$Neu5NAc_1Hex_8HexNAc_5$	3288.6	3288.6	2656.9	2656.8
$OAc_1Neu5NAc_1Hex_8HexNAc_5$			2699.0	2698.7
$Neu5NAc_2Hex_7HexNAc_5$	3445.7	3445.6	2799.0	2798.8
$OAc_1Neu5NAc_2Hex_7HexNAc_5$			2841.0	2840.8
$OAc_2Neu5NAc_2Hex_7HexNAc_5$			2883.0	2882.8
$Neu5NAc_1Hex_9HexNAc_6$	3737.9	3737.8	3022.1	3022.9
$Neu5NAc_1Hex_{10}HexNAc_6$	3942.0	3941.8	3184.1	3184.9
$Neu5NAc_2Hex_9HexNAc_6$	4099.0	4098.9	3326.2	3326.0
$OAc_1Neu5NAc_2Hex_9HexNAc_6$			3368.2	3368.9
$OAc_2Neu5NAc_2Hex_9HexNAc_6$			3410.2	3411.0
$Neu5NAc_3Hex_9HexNAc_6$	4460.2	4460.1	3630.3	3630.0
$OAc_1Neu5NAc_3Hex_9HexNAc_6$			3672.3	3672.0
$OAc_2Neu5NAc_3Hex_9HexNAc_6$			3714.4	3714.0
$OAc_3Neu5NAc_3Hex_9HexNAc_6$			3756.4	3757.8

* Monoisotopic mass units were used for calculation of molecular mass based on proposed composition.
** Not detected.

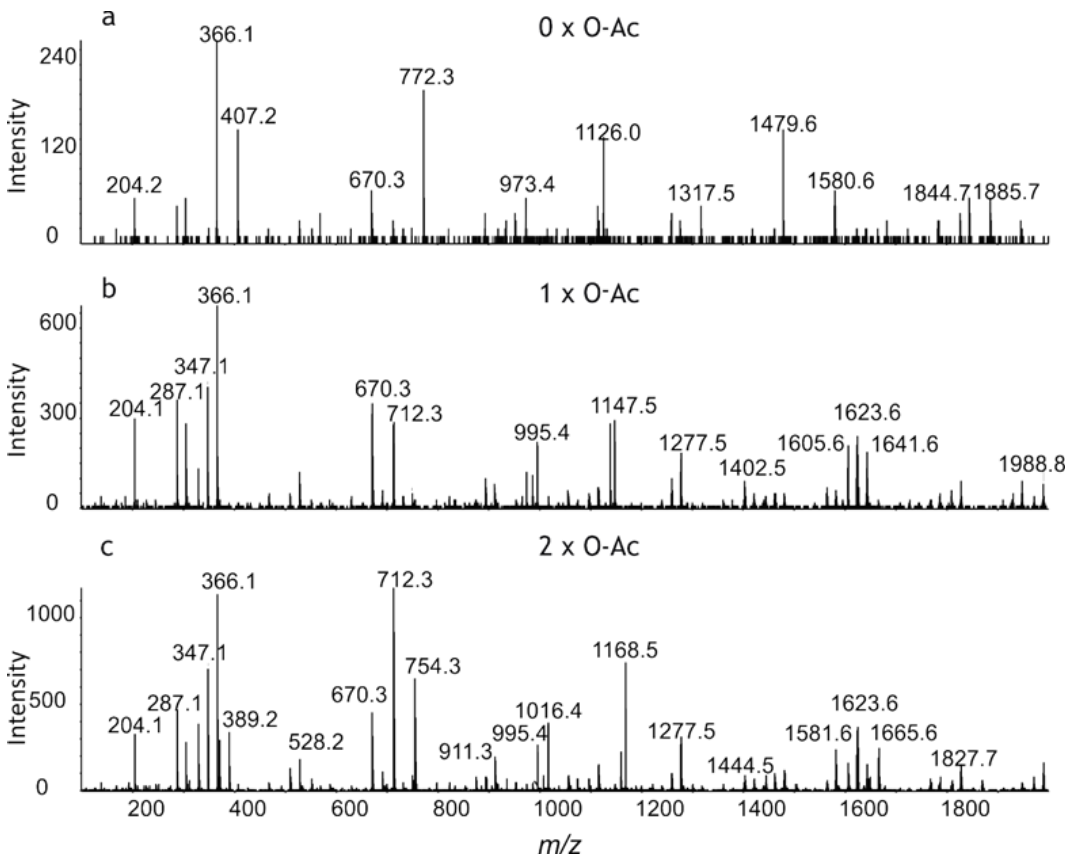

Figure S7. LC-MS/MS analysis of methylamidated N-glycans isolated from a representative serum sample of silver carp (Silver-1). (a) MS/MS spectrum of $[M+2H]^{2+}$ at *m/z* 1125.4 ($Neu5NAc_2Hex_5HexNAc_4$); (b) MS/MS spectrum of $[M+2H]^{2+}$ at *m/z* 1146.4 ($OAc_1Neu5NAc_2Hex_5HexNAc_4$); (c) MS/MS spectrum of $[M+2H]^{2+}$ at *m/z* 1167.5 ($OAc_2Neu5NAc_2Hex_5HexNAc_4$).

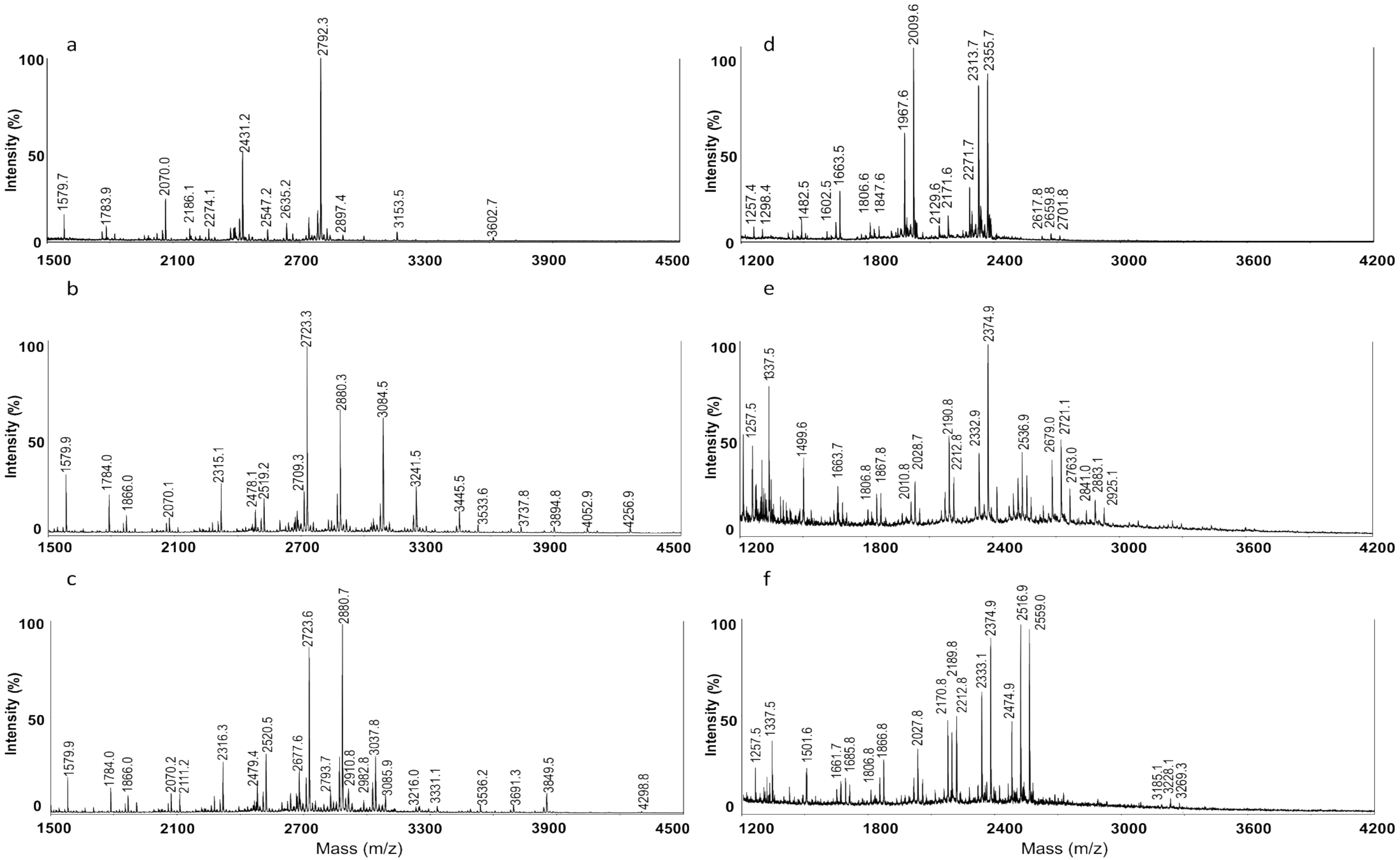

Figure S8. MALDI-MS analysis of N-glycans isolated from three serum sample replicates of silver carp, labelled as Silver-1, Silver-2, Silver-3: (a) permethylated glycans from Silver-1 (see also Figure 5a); (b) permethylated glycans from Silver-2; (c) permethylated glycans from Silver-3; (d) methylamidated glycans from Silver-1 (see also Figure 5b); (e) methylamidated glycans from Silver-2; (f) methylamidated glycans from Silver-3. The ions at *m/z* 1337.5, *m/z* 1499.6 and *m/z* 1661.7 appear to be associated with hexose polymers, *i.e.* $[M+Na]^+$ of Hex_8, Hex_9 and Hex_{10}, respectively. These background ions possibly originated from the cellulose SPE.

Table S4. Detected ions and their corresponding compositions of N-glycans from silver carp serum samples

Proposed composition	Permethylation		Methylamidation	
	Theoretical* [M+Na]+ (*m/z*)	Detected [M+Na]+ (*m/z*)	Theoretical [M+Na]+ (*m/z*)	Detected [M+Na]+ (*m/z*)
$Hex_5HexNAc_2$	1579.8	1579.8	1257.4	1257.6
$Hex_4HexNAc_3$	1620.8	1620.8	1298.5	1298.4
$Hex_6HexNAc_2$	1783.9	1783.9	1419.5	1419.4
$Neu5NAc_1Hex_4HexNAc_3$	1982.0	1982.0	1602.6	1602.5
$OAc_1Neu5NAc_1Hex_4HexNAc_3$			1644.6	1644.5
$Hex_5HexNAc_4$	2070.0	2070.0	1663.6	1663.5
$Neu5NAc_1Hex_4HexNAc_4$	2227.1	2227.1	1805.7	1805.6
$OAc_1Neu5NAc_1Hex_4HexNAc_4$			1847.7	1847.6
$Hex_6HexNAc_4$	2274.1	2274.1	1825.6	1825.5
$Neu5NAc_1Hex_5HexNAc_4$	2431.2	2431.2	1967.7	1967.6
$OAc_1Neu5NAc_1Hex_5HexNAc_4$			2009.7	2009.6
$Neu5NAc_1Hex_6HexNAc_4$	2635.3	2635.2	2129.8	2129.6
$OAc_1Neu5NAc_1Hex_6HexNAc_4$			2171.8	2171.6
$Hex_7HexNAc_5$	2723.4	2723.3	2190.8	2190.8
$Neu5NAc_1Hex_6HexNAc_5$	2880.4	2880.3**	2332.8	2332.9
$OAc_1Neu5NAc_1Hex_6HexNAc_5$			2374.9	2374.9
$Neu5NAc_2Hex_5HexNAc_4$	2792.4	2792.3	2271.8	2271.7
$OAc_1Neu5NAc_2Hex_5HexNAc_4$			2313.9	2313.7
$OAc_2Neu5NAc_2Hex_5HexNAc_4$			2355.9	2355.7
$Neu5NAc_2Hex_5HexNAc_5$	3037.5	3037.8**	2474.9	2474.9
$OAc_1Neu5NAc_2Hex_5HexNAc_5$			2516.9	2516.9
$OAc_2Neu5NAc_2Hex_5HexNAc_5$			2558.9	2559.0
$OAc_1Neu5NAc_1Hex_7HexNAc_5$	3084.5	3084.5	2536.9	2536.9
$OAc_1Neu5NAc_2Hex_6HexNAc_5$	3241.6	3241.5	2679.0	2679.0
$OAc_2Neu5NAc_2Hex_6HexNAc_5$			2721.0	2721.1
$OAc_3Neu5NAc_2Hex_6HexNAc_5$			2763.0	2763.0
$OAc_1Neu5NAc_2Hex_7HexNAc_5$	3445.7	3445.5	2841.0	2841.0
$OAc_2Neu5NAc_2Hex_7HexNAc_5$			2883.0	2883.1
$OAc_3Neu5NAc_2Hex_7HexNAc_5$			2925.1	2925.1
$Neu5NAc_3Hex_6HexNAc_5$	3602.8	3602.7	2941.1	2941.0
$OAc_1Neu5NAc_3Hex_6HexNAc_5$			2983.1	2983.0
$OAc_2Neu5NAc_3Hex_6HexNAc_5$			3025.1	3024.9

* Monoisotopic mass units were used for calculation of molecular mass based on proposed composition.
**detected in Silver-2 and Silver-3.

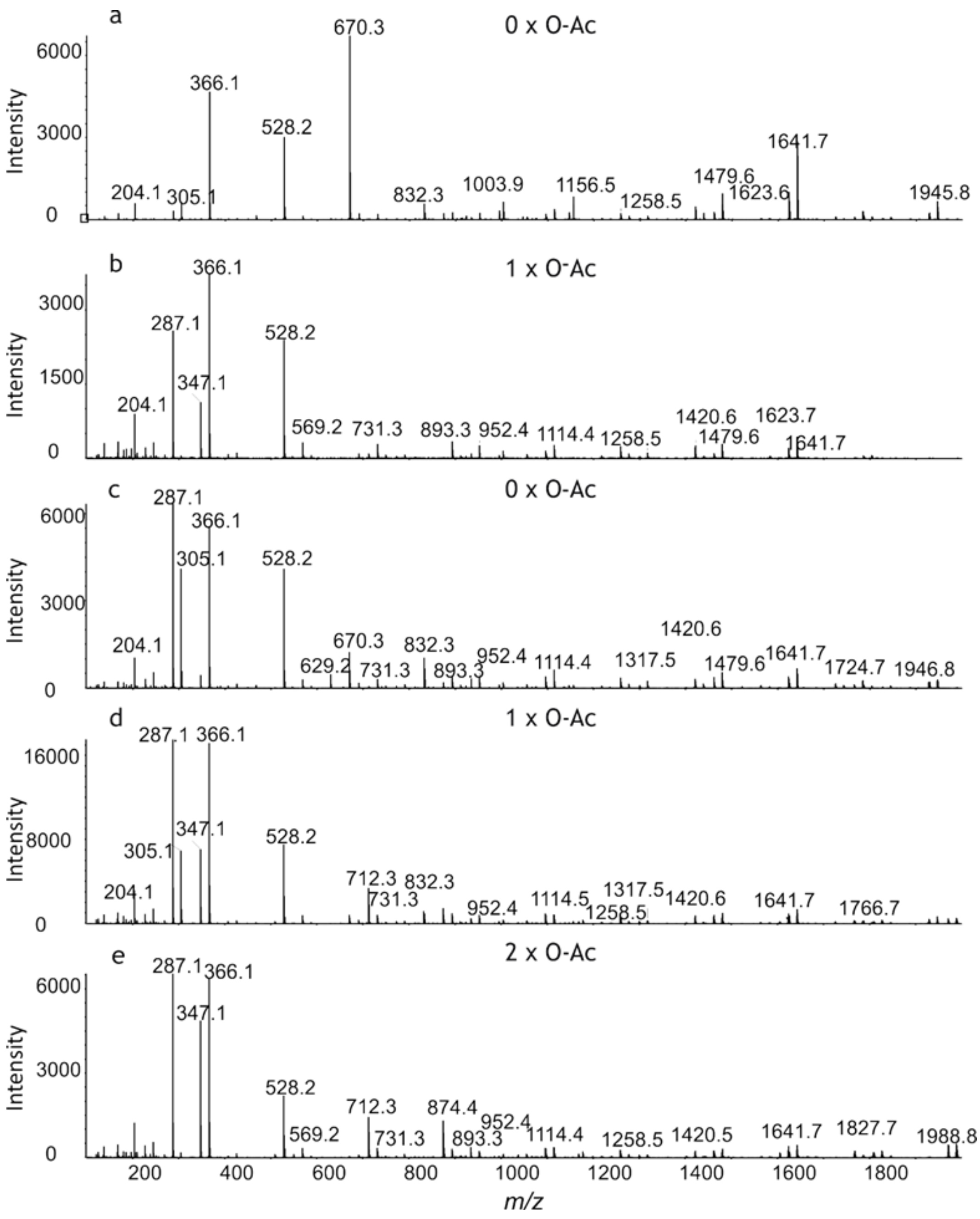

Figure S9. LC-MS/MS analysis of methylamidated N-glycans isolated from a representative serum sample of bream carp (Bream-1). (a) MS/MS spectrum of $[M+2H]^{2+}$ at *m/z* 1156.0 ($Neu5NAc_1Hex_6HexNAc_5$); (b) MS/MS spectrum of $[M+3H]^{3+}$ at *m/z* 785.0 ($OAc_1Neu5NAc_1Hex_6HexNAc_5$); (c) MS/MS spectrum of $[M+3H]^{3+}$ at *m/z* 872.4 ($Neu5NAc_2Hex_6HexNAc_5$); (d) MS/MS spectrum of $[M+3H]^{3+}$ at *m/z* 886.4 ($OAc_1Neu5NAc_2Hex_6HexNAc_5$); (e) MS/MS spectrum of $[M+3H]^{3+}$ at *m/z* 900.4 ($OAc_2Neu5NAc_2Hex_6HexNAc_5$).

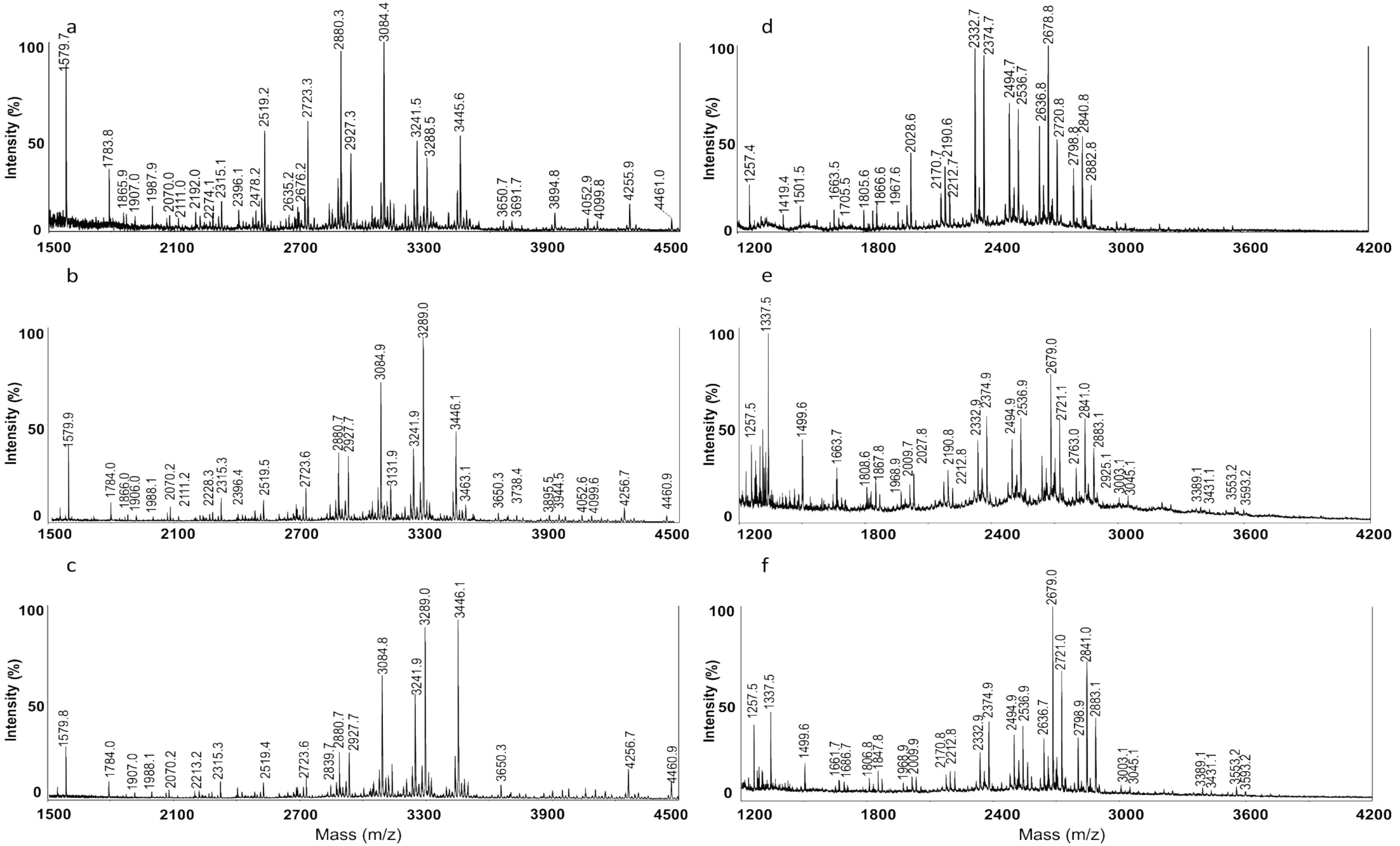

Figure S10. MALDI-MS analysis of N-glycans isolated from three serum sample replicates of bream carp, labelled as Bream-1, Bream-2, Bream-3: (a) permethylated glycans from Bream-1 (see also Figure 6a); (b) permethylated glycans from Bream-2; (c) permethylated glycans from Bream-3; (d) methylamidated glycans from Bream-1 (see also Figure 6b); (e) methylamidated glycans from Bream-2; (f) methylamidated glycans from Bream-3. The ions at *m/z* 1337.5, *m/z* 1499.6 and *m/z* 1661.7 appear to be associated with hexose polymers, *i.e.* $[M+Na]^+$ of Hex_8, Hex_9 and Hex_{10}, respectively. These background ions possibly originated from the cellulose SPE.

Table S5. Detected ions and their corresponding compositions of N-glycans from bream serum samples

**Proposed composition**	**Permethylation**		**Methylamidation**	
	Theoretical* [M+Na]$^+$ (*m/z*)	Detected [M+Na]$^+$ (*m/z*)	Theoretical [M+Na]$^+$ (*m/z*)	Detected [M+Na]$^+$ (*m/z*)
Hex$_5$HexNAc$_2$	1579.8	1579.7	1257.4	1257.4
Hex$_6$HexNAc$_2$	1783.9	1783.8	1419.5	1419.4
Hex$_4$HexNAc$_4$	1865.9	1865.9	1501.5	1501.5
Hex$_3$HexNAc$_5$	1907.0	1907.0	1542.6	1541.4
Hex$_7$HexNAc$_2$	1988.0	1987.9	1581.5	1581.4
Hex$_5$HexNAc$_4$	2070.0	2070.0	1663.6	1663.5
Hex$_4$HexNAc$_5$	2111.1	2111.0	1704.6	1705.5
Hex$_8$HexNAc$_2$	2192.1	2192.0	1743.6	1743.5
Neu5NAc$_1$Hex$_4$HexNAc$_4$	2227.1	2227.1	1805.7	1805.6
OAc$_1$Neu5NAc$_1$Hex$_4$HexNAc$_4$			1847.7	1847.5
Hex$_6$HexNAc$_4$	2274.1	2274.1	1825.6	1825.5
Hex$_5$HexNAc$_5$	2315.2	2315.1	1866.7	1866.6
Hex$_9$HexNAc$_2$	2396.2	2396.1	1905.6	1905.5
Neu5NAc$_1$Hex$_5$HexNAc$_4$	2431.2	2431.1	1967.7	1967.6
OAc$_1$Neu5NAc$_1$Hex$_5$HexNAc$_4$			2009.7	2009.6
Hex$_7$HexNAc$_4$	2478.2	2478.2	1987.7	1987.6
Hex$_6$HexNAc$_5$	2519.3		2028.7	2170.7
Neu5NAc$_2$He$_5$HexNAc$_3$	2547.3	2519.2	2068.8	2028.6
Neu5NAc$_1$Hex$_5$HexNAc$_5$	2676.3	2676.2	2170.8	2170.7
OAc$_1$Neu5NAc$_1$Hex$_5$HexNAc$_5$			2212.8	2212.7
Hex$_7$HexNAc$_5$	2723.4	2723.3	2190.8	2190.6
Neu5NAc$_1$Hex$_6$HexNAc$_5$	2880.4	2880.3	2332.8	2332.7
OAc$_1$Neu5NAc$_1$Hex$_6$HexNAc$_5$			2374.9	2374.7
Hex$_8$HexNAc$_5$	2927.5	2927.3	2352.8	2352.7
Neu5NAc$_1$Hex$_7$HexNAc$_5$	3084.5	3084.4	2494.9	2494.7
OAc$_1$Neu5NAc$_1$Hex$_7$HexNAc$_5$			2536.9	2536.7
Neu5NAc$_2$Hex$_6$HexNAc$_5$	3241.6	3241.5	2637.0	2636.8
OAc$_1$Neu5NAc$_2$Hex$_6$HexNAc$_5$			2679.0	2678.8
OAc$_2$Neu5NAc$_2$Hex$_6$HexNAc$_5$			2721.0	2720.8
Neu5NAc$_1$Hex$_8$HexNAc$_5$	3288.6	3288.5	2656.9	2656.7
Neu5NAc$_1$Hex$_7$HexNAc$_6$	3329.7	3329.5	2698.0	2697.7
OAc$_1$Neu5NAc$_1$Hex$_7$HexNAc$_6$			2740.0	2739.8
Neu5NAc$_2$Hex$_7$HexNAc$_5$	3445.7	3445.6	2799.0	2798.8
OAc$_1$Neu5NAc$_2$Hex$_7$HexNAc$_5$			2841.0	2840.8
OAc$_2$Neu5NAc$_2$Hex$_7$HexNAc$_5$			2883.0	2882.8
Neu5NAc$_3$Hex$_6$HexNAc$_5$	3602.8	3602.5	2941.1	2941.8
Neu5NAc$_2$Hex$_8$HexNAc$_5$	3649.8	3650.7	2961.1	2960.8
Neu5NAc$_2$Hex$_7$HexNAc$_6$	3690.8	3691.7	3002.1	3002.9

$Neu5NAc_2Hex_8HexNAc_6$	3894.9	3894.8	3164.2	3163.9
$OAc_1Neu5NAc_2Hex_8HexNAc_6$			3206.2	3207.8
$OAc_2Neu5NAc_2Hex_8HexNAc_6$			3248.2	3247.9
$Neu5NAc_3Hex_6HexNAc_7$	4093.0	4092.8	3347.3	ND**
$OAc_1Neu5NAc_3Hex_6HexNAc_7$			3389.3	3391.0
$OAc_2Neu5NAc_3Hex_6HexNAc_7$			3431.3	3431.8
$Neu5NAc_2Hex_9HexNAc_6$	4099.0	4099.8	3326.2	ND
$OAc_1Neu5NAc_2Hex_9HexNAc_6$			3368.2	3368.7
$OAc_2Neu5NAc_2Hex_9HexNAc_6$			3410.2	3410.7
$Neu5NAc_3Hex_8HexNAc_6$	4256.1	4255.9	3468.3	ND
$OAc_1Neu5NAc_3Hex_8HexNAc_6$			3510.3	3510.1
$OAc_2Neu5NAc_3Hex_8HexNAc_6$			3552.3	3552.0
$OAc_3Neu5NAc_3Hex_8HexNAc_6$			3594.3	3595.4
$Neu5NAc_3Hex_9HexNAc_6$	4460.2	4461.0	3630.3	ND

* Monoisotopic mass units were used for calculation of molecular mass based on proposed composition.
** Not detected.

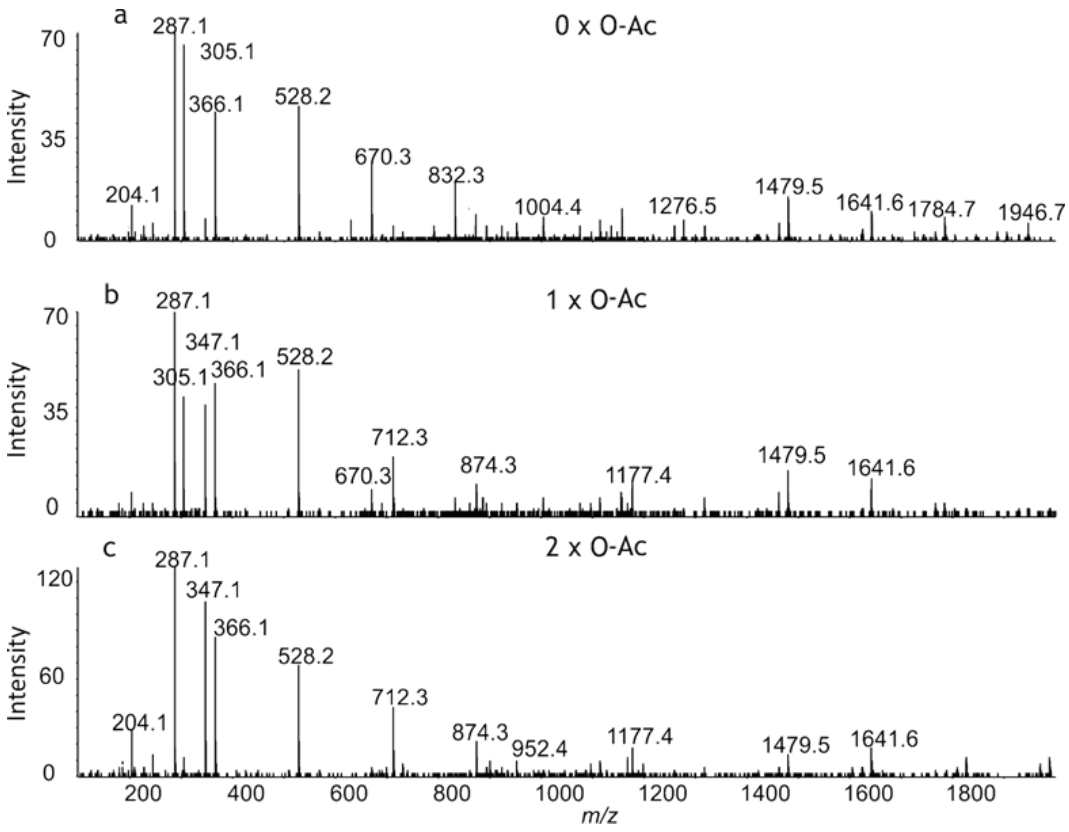

Figure S11. LC-MS/MS analysis of methylamidated *N*-glycans isolated from a representative serum sample of bighead carp (Bighead-1). (a) MS/MS spectrum of $[M+3H]^{3+}$ at *m/z* 872.3 ($Neu5NAc_2Hex_6HexNAc_5$); (b) MS/MS spectrum of $[M+3H]^{3+}$ at *m/z* 886.3 ($OAc_1Neu5NAc_2Hex_6HexNAc_5$); (c) MS/MS spectrum of $[M+3H]^{3+}$ at *m/z* 900.3 ($OAc_2Neu5NAc_2Hex_6HexNAc_5$).

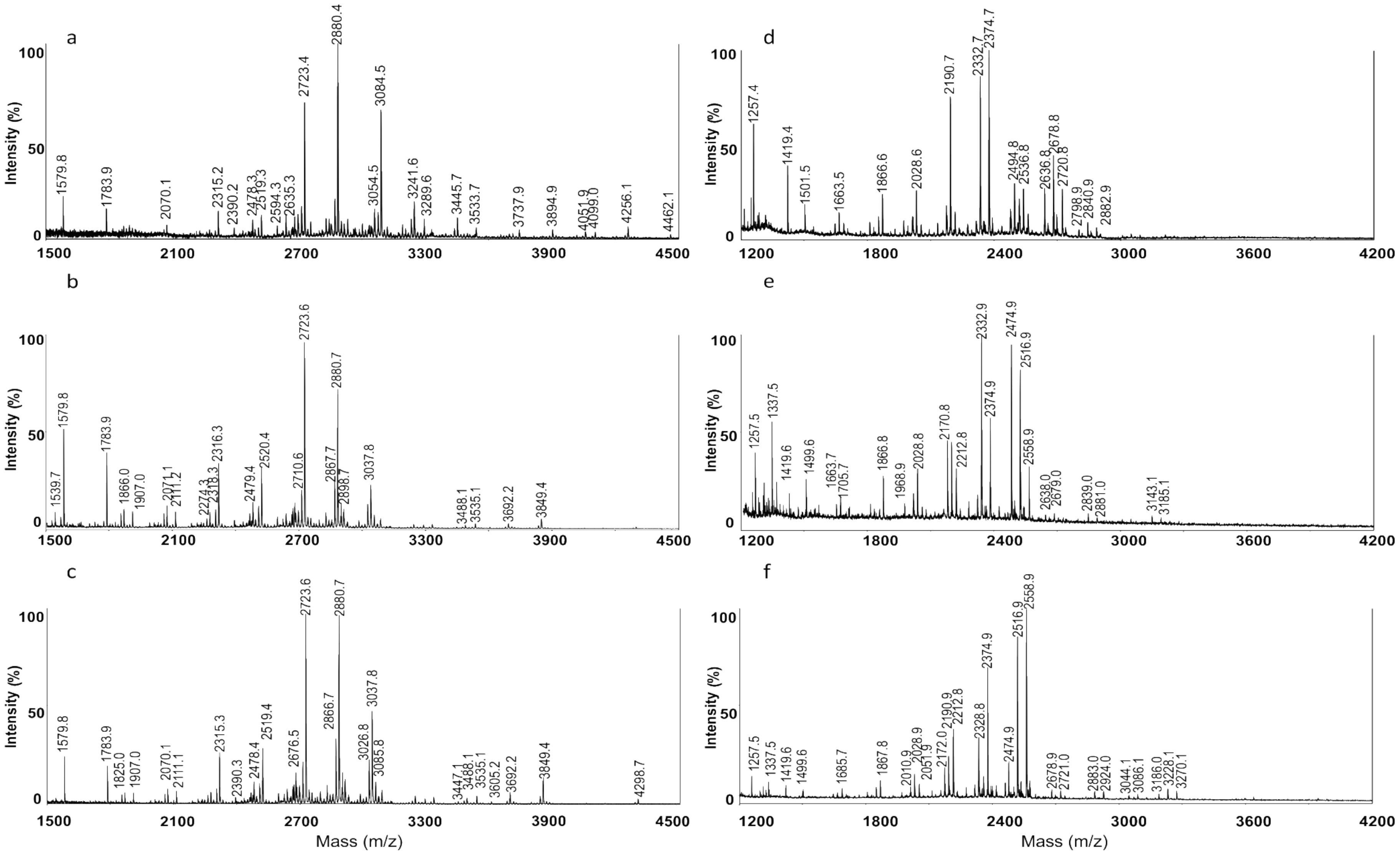

Figure S12. MALDI-MS analysis of N-glycans isolated from three serum sample replicates of bighead carp, labelled as Bighead-1, Bighead-2, Bighead-3: (a) permethylated glycans from Bighead-1 (see also Figure 7a); (b) permethylated glycans from Bighead-2; (c) permethylated glycans from Bighead-3; (d) methylamidated glycans from Bighead-1 (see also Figure 7b); (e) methylamidated glycans from Bighead-2; (f) methylamidated glycans from Bighead-3. The ions at *m/z* 1337.5 and *m/z* 1499.6 appear to be associated with hexose polymers, *i.e.* $[M+Na]^+$ of Hex_8 and Hex_9, respectively. These background ions possibly originated from the cellulose SPE.

Table S6. Detected ions and their corresponding compositions of N-glycans from bighead carp serum

Proposed composition	Permethylation		Methylamidation	
	Theoretical* $[M+Na]^+$ (*m/z*)	Detected $[M+Na]^+$ (*m/z*)	Theoretical $[M+Na]^+$ (*m/z*)	Detected $[M+Na]^+$ (*m/z*)
$Hex_5HexNAc_2$	1579.8	1579.8	1257.4	1257.4
$Hex_6HexNAc_2$	1783.9	1783.9	1419.5	1419.4
$Hex_4HexNAc_4$	1865.9	1866.0	1501.5	1501.5
$Hex_3HexNAc_5$	1907.0	1906.2	1542.6	1542.5
$Hex_5HexNAc_4$	2070.0	2070.1	1663.6	1663.5
$Hex_5HexNAc_5$	2315.2	2315.2	1866.7	1866.6
$Neu5NAc_1Hex_5HexNAc_4$	2431.2	2431.3	1967.7	1967.6
$OAc_1Neu5NAc_1Hex_5HexNAc_4$			2009.7	2009.6
$OAc_2Neu5NAc_1Hex_5HexNAc_4$			2051.7	2051.7
$Hex_7HexNAc_4$	2478.2	2478.3	1987.7	1987.6
$Hex_6HexNAc_5$	2519.3	2519.3	2028.7	2028.6
$Neu5NAc_1Hex_6HexNAc_4$	2635.3	2635.2	2129.8	2129.7
$OAc_1Neu5NAc_1Hex_6HexNAc_4$			2171.8	2171.7
$Hex_7HexNAc_5$	2723.4	2723.4	2190.8	2190.7
$Neu5NAc_1Hex_6HexNAc_5$	2880.4	2880.4	2332.8	2332.7
$OAc_1Neu5NAc_1Hex_6HexNAc_5$			2374.9	2374.7
$Neu5NAc_2Hex_5HexNAc_5$	3037.5	3037.8	2474.9	2474.9
$OAc_1Neu5NAc_2Hex_5HexNAc_5$			2516.9	2516.9
$OAc_2Neu5NAc_2Hex_5HexNAc_5$			2558.9	2558.9
$Neu5NAc_1Hex_7HexNAc_5$	3084.5	3084.4	2494.9	2494.8
$OAc_1Neu5NAc_1Hex_7HexNAc_5$			2536.9	2536.8
$Hex_9HexNAc_5$	3131.6	3131.5	2514.9	2514.8
$Neu5NAc_2Hex_6HexNAc_5$	3241.6	3241.6	2637.0	2636.8
$OAc_1Neu5NAc_2Hex_6HexNAc_5$			2679.0	2678.8
$OAc_2Neu5NAc_2Hex_6HexNAc_5$			2721.0	2720.8
$Neu5NAc_2Hex_7HexNAc_5$	3445.7	3445.7	2799.0	2798.9
$OAc_1Neu5NAc_2Hex_7HexNAc_5$			2841.0	2840.9
$OAc_2Neu5NAc_2Hex_7HexNAc_5$			2883.0	2882.9
$Neu5NAc_1Hex_8HexNAc_6$	3533.8	3533.7	2860.0	2860.8
$Neu5NAc_2Hex_7HexNAc_6$	3690.8	3690.8	3002.1	3002.9
$OAc_1Neu5NAc_2Hex_7HexNAc_6$			3044.1	3044.9
$OAc_2Neu5NAc_2Hex_7HexNAc_6$			3086.1	3087.0
$Neu5NAc_2Hex_8HexNAc_6$	3894.9	3894.9	3164.2	3163.9
$Neu5NAc_3Hex_8HexNAc_5$	4011.0	4012.1	3265.2	3265.0
$Neu5NAc_3Hex_7HexNAc_6$	4052.0	4051.9	3306.2	3360.6
$OAc_1Neu5NAc_3Hex_7HexNAc_6$			3348.2	3348.8
$OAc_2Neu5NAc_3Hex_7HexNAc_6$			3086.1	3087.0

* Monoisotopic mass units were used for calculation of molecular mass based on proposed composition.

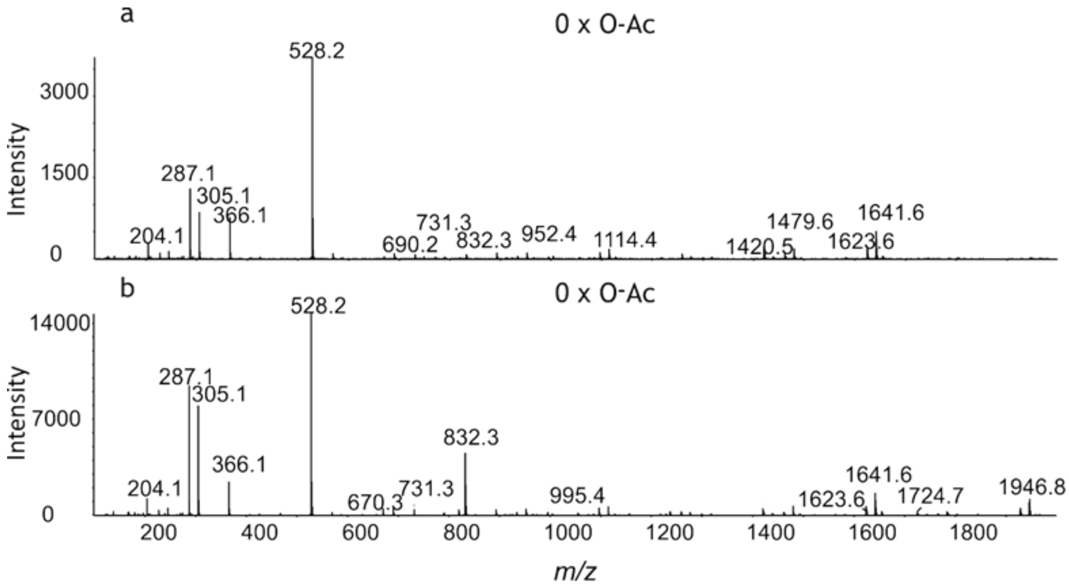

Figure S13. LC-MS/MS analysis of methylamidated N-glycans isolated from a representative serum sample of black carp (Black-1). (a) MS/MS spectrum of $[M+3H]^{3+}$ at *m/z* 824.9 ($Neu5NAc_1Hex_7HexNAc_5$); (b) MS/MS spectrum of $[M+3H]^{3+}$ at *m/z* 926.4 ($Neu5NAc_2Hex_7HexNAc_5$).

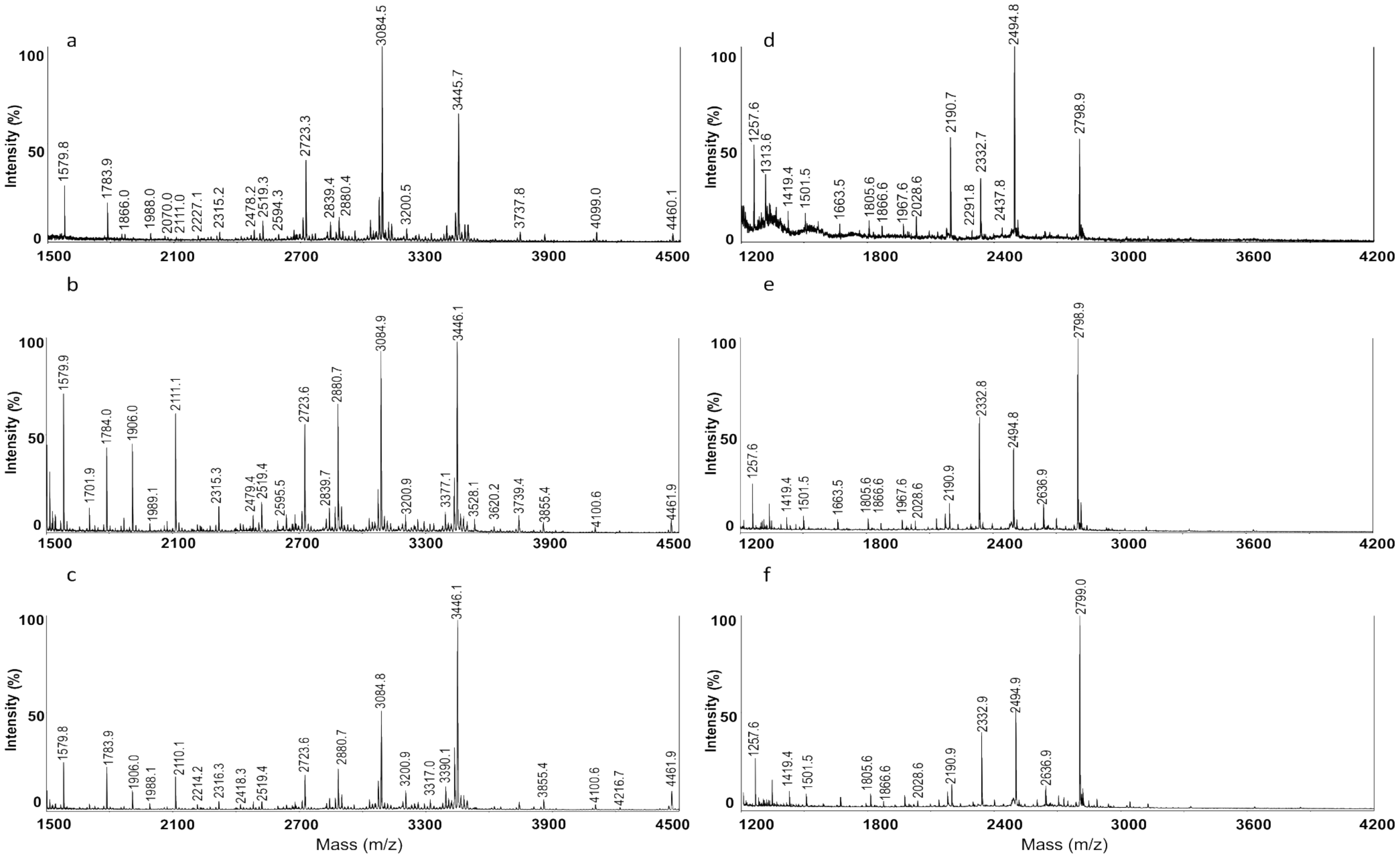

Figure S14. MALDI-MS analysis of N-glycans isolated from three serum sample replicates of black carp, Black-1, Black-2, Black-3: (a) permethylated glycans from Black-1 (see also Figure 8a); (b) permethylated glycans from Black-2; (c) permethylated glycans from Black-3; (d) methylamidated glycans from Black-1 (see also Figure 8b); (e) methylamidated glycans from Black-2; (f) methylamidated glycans from Black-3.

Table S7. Detected ions and their corresponding compositions of N-glycans from black carp serum samples

**Proposed composition**	**Permethylation**		**Methylamidation**	
	Theoretical* $[M+Na]^+$ (*m/z*)	Detected $[M+Na]^+$ (*m/z*)	Theoretical $[M+Na]^+$ (*m/z*)	Detected $[M+Na]^+$ (*m/z*)
$Hex_5HexNAc_2$	1579.8	1579.8	1257.4	1257.4
$Hex_6HexNAc_2$	1783.9	1783.9	1419.5	1419.4
$Hex_4HexNAc_4$	1865.9	1865.9	1501.5	1501.5
$Hex_7HexNAc_2$	1988.0	1988.0	1581.5	ND**
$Hex_5HexNAc_4$	2070.0	2070.1	1663.6	1663.5
$Neu5NAc_1Hex_4HexNAc_4$	2227.1	2227.1	1805.7	1805.6
$Hex_5HexNAc_5$	2315.2	2315.2	1866.7	1866.6
$Neu5NAc_1Hex_5HexNAc_4$	2431.2	2431.2	1967.7	1967.6
$Hex_7HexNAc_4$	2478.2	2478.2	1987.7	1987.6
$Hex_6HexNAc_5$	2519.3	2519.3	2028.7	2086.0
$Hex_7HexNAc_5$	2723.4	2723.4	2190.8	2190.7
$Neu5NAc_1Hex_7HexNAc_4$	2839.4	2839.4	2291.8	2291.8
$Neu5NAc_1Hex_6HexNAc_5$	2880.4	2880.4	2332.8	2332.7
$Neu5NAc_1Hex_7HexNAc_5$	3084.5	3084.5	2494.9	2494.8
$Neu5NAc_2Hex_7HexNAc_5$	3445.7	3445.7	2799.0	2798.9
$Neu5NAc_1Hex_9HexNAc_6$	3737.9	3737.8	3022.1	3022.9
$Neu5NAc_2Hex_9HexNAc_6$	4099.0	4099.0	3326.2	3326.9

* Monoisotopic mass units were used for calculation of molecular mass based on proposed composition.
** Not detected.
